# Comparing Linkage Designs Based on Land Facets to Linkage Designs Based on Focal Species

**DOI:** 10.1371/journal.pone.0048965

**Published:** 2012-11-12

**Authors:** Brian M. Brost, Paul Beier

**Affiliations:** School of Forestry and Merriam-Powell Center for Environmental Research, Northern Arizona University, Flagstaff, Arizona, United States of America; University of Western Ontario, Canada

## Abstract

Least-cost modeling for focal species is the most widely used method for designing conservation corridors and linkages. However, these designs depend on today's land covers, which will be altered by climate change. We recently proposed an alternative approach based on land facets (recurring landscape units of relatively uniform topography and soils). The rationale is that corridors with high continuity of individual land facets will facilitate movement of species associated with each facet today and in the future. Conservation practitioners might like to know whether a linkage design based on land facets is likely to provide continuity of modeled breeding habitat for species needing connectivity today, and whether a linkage for focal species provides continuity and interspersion of land facets. To address these questions, we compared linkages designed for focal species and land facets in three landscapes in Arizona, USA. We used two variables to measure linkage utility, namely distances between patches of modeled breeding habitat for 5–16 focal species in each linkage, and resistance profiles for focal species and land facets between patches connected by the linkage. Compared to focal species designs, linkage designs based on land facets provided as much or more modeled habitat connectivity for 25 of 28 species-landscape combinations, failing only for the three species with the most narrowly distributed habitat. Compared to land facets designs, focal species linkages provided lower connectivity for about half the land facets in two landscapes. In areas where a focal species approach to linkage design is not possible, our results suggest that conservation practitioners may be able to implement a land facets approach with some confidence that the linkage design would serve most potential focal species. In areas where focal species designs are possible, we recommend using the land facet approach to complement, rather than replace, focal species approaches.

## Introduction

**Figure 1:**
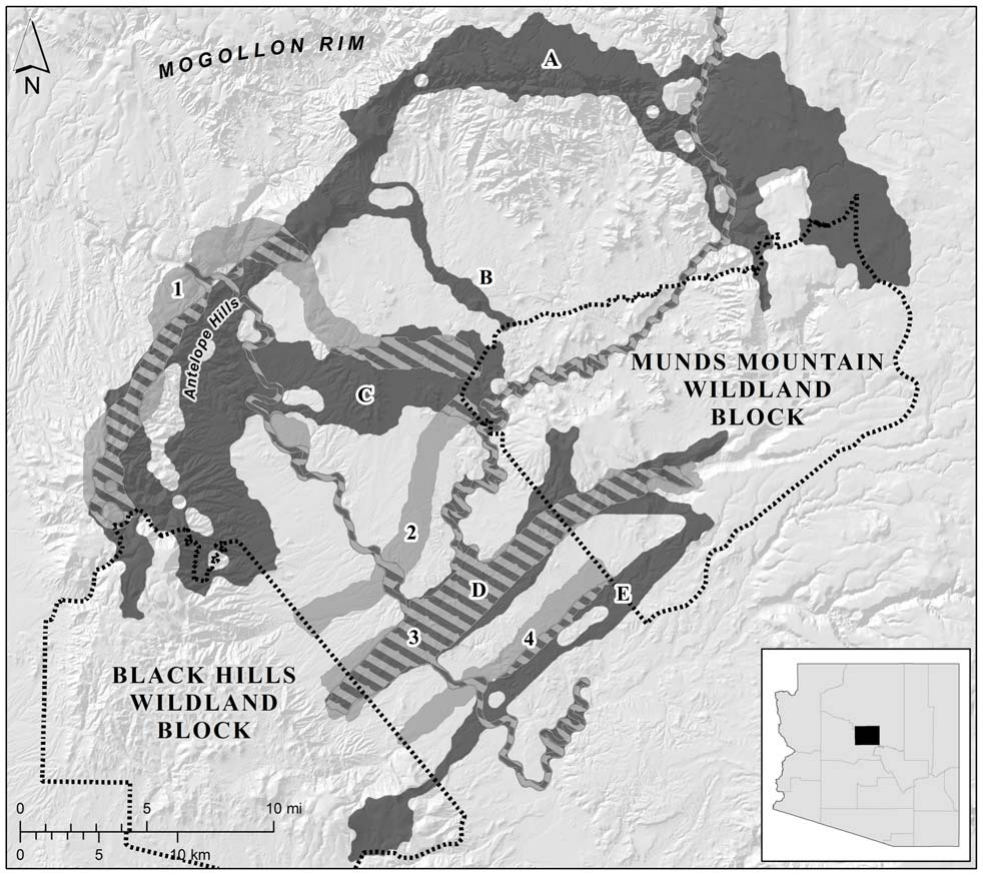
Map of the linkage designs for the Black Hills-Munds Mountain planning area. The land facets design is shown in dark grey and the focal species design is shown in light grey; hatching indicates where the two types of linkage designs overlap. Linkage strands in the land facets design consisted of corridors for (A) high elevation, gentle canyon bottoms and ridges; and high elevation, gentle, hot slopes; (B) high diversity of land facets; (C) low elevation, gentle, canyon bottoms and ridges; and low elevation, gentle, warm slopes; (D) mid elevation, steep canyon bottoms and ridges; low elevation, steep, cool slopes; and mid elevation, steep, warm slopes; and (E) mid elevation, gentle, warm slopes. Linkage strands in the focal species design consisted of corridors for (1) elk, (2) mule deer, (3) black bear, and (4) javelina. Although a corridor was not designed explicitly for mountain lion, linkage strands 1 and 2 contained large amounts of optimal or suitable habitat for this species. Inset shows location within Arizona, USA.

Designing and protecting conservation corridors and linkages is one strategy to conserve connectivity in landscapes increasingly dominated by human activities [1], [2]. They are also the most commonly recommended strategy for biodiversity management in the face of climate change [3]. Whereas individual linkages support relatively short-term movements between neighboring wildlands (e.g., Figure 1), a network of linkages connecting multiple wildlands can facilitate long-term range shift by accommodating repeated movements of species tracking changes in climate. Least-cost modeling for focal species is the most widely used method for designing corridors to connect existing wildland blocks (e.g., 23 studies listed in [2]).

Least-cost modeling attempts to identify the swath of land (i.e., corridor) that minimizes species-specific resistance to movement between two termini (wildland blocks) [2], [4]. Resistance is a function of cell attributes in a geographic information system and is usually estimated as the inverse of habitat quality [2]. Corridors for multiple focal species are combined into a preliminary linkage design, which becomes the final linkage design upon modification to accommodate ecological processes, non-modeled species, or edge effects [2].

Like most other conservation plans, least-cost corridors have been designed given the present distributions of focal species and are largely based on present land cover; however, land cover—and focal species' distributions—will change as climate changes [5]. Thus, it is uncertain how well these linkages will function when some species currently occupying an area no longer do so and other species arrive.

An alternative, coarse-filter approach is to base conservation plans on physical environments because they are more stable with respect to climate change [5]–[8]. Brost and Beier [9] applied this approach to linkage design by classifying a landscape into land facets, or recurring areas of relatively homogenous topography and soils, and designing a linkage to optimize their connectivity and interspersion. This strategy operates on the premise that diverse physical environments support diverse species [10]–[14] and the ecological and evolutionary processes that maintain and generate biodiversity [13], [15]–[21]. Thus, a linkage designed to provide continuity for all land facets should optimize connectivity for the full diversity of plants and animals and sustain these processes.

Brost and Beier [9] provide procedures to design linkages based on land facets. Each linkage includes multiple partially-overlapping corridors, resulting in a multi-stranded linkage design (e.g., Figure 1). In each linkage design, one corridor optimizes connectivity for high interspersion (local diversity) of land facets; this corridor is intended to accommodate rapid, short-distance (intra-corridor) movements between different land facets (e.g., low to high elevation or warm to cool aspects), interactions between species, and ecological and evolutionary processes that depend on interactions [20], [21]. Each of the other corridors optimizes connectivity for one facet type, and is intended to facilitate movement of species associated with that facet, today and in the future. Beier and Brost [8] developed their approach for landscapes in the Western U.S., where large (>350 km^2^) wildland blocks containing high diversity of land facets, juxtaposed in complex ways, are typically separated by ∼10–30 km. In this context, transitions from warmer land facets (low elevation, antipodal aspects) to cooler ones can best occur within the wildland blocks; thus their designs did not include additional “directional corridors” to connect warm facets in each wildland to cooler land facets in the other wildland.

Although Beier and Brost [8] and Brost and Beier [9] recommend using land facets in conjunction with focal species to design linkages, conservation practitioners may be limited to a land facets approach in areas where species information is poor or where land cover maps do not exist. Such practitioners might like to know whether a linkage design based on land facets is likely to support movement by local species needing connectivity. Practitioners who have designed a linkage for focal species might also want to know if the design provides for continuity and interspersion of land facets, or whether additional land should be conserved to better capture physical environments. Practitioners may also want to know how much area is required for each type of linkage design.

In this paper we consider how well linkages based on land facets provide connectivity of modeled habitat for focal species, and how well linkages designed for focal species provide continuity of land facets. If diverse physical environments support diverse species, linkages designed using land facets should serve species not only in the future, but also today. Conversely, linkages designed for diverse focal species should also contain diverse physical environments because many focal species corridors are produced by models that optimize continuity of land covers (partially determined by physical environment) and topographic elements [2]. Although both expectations are reasonable, this is the first paper to examine the issue in the context of linkage design.

**Figure 2:**
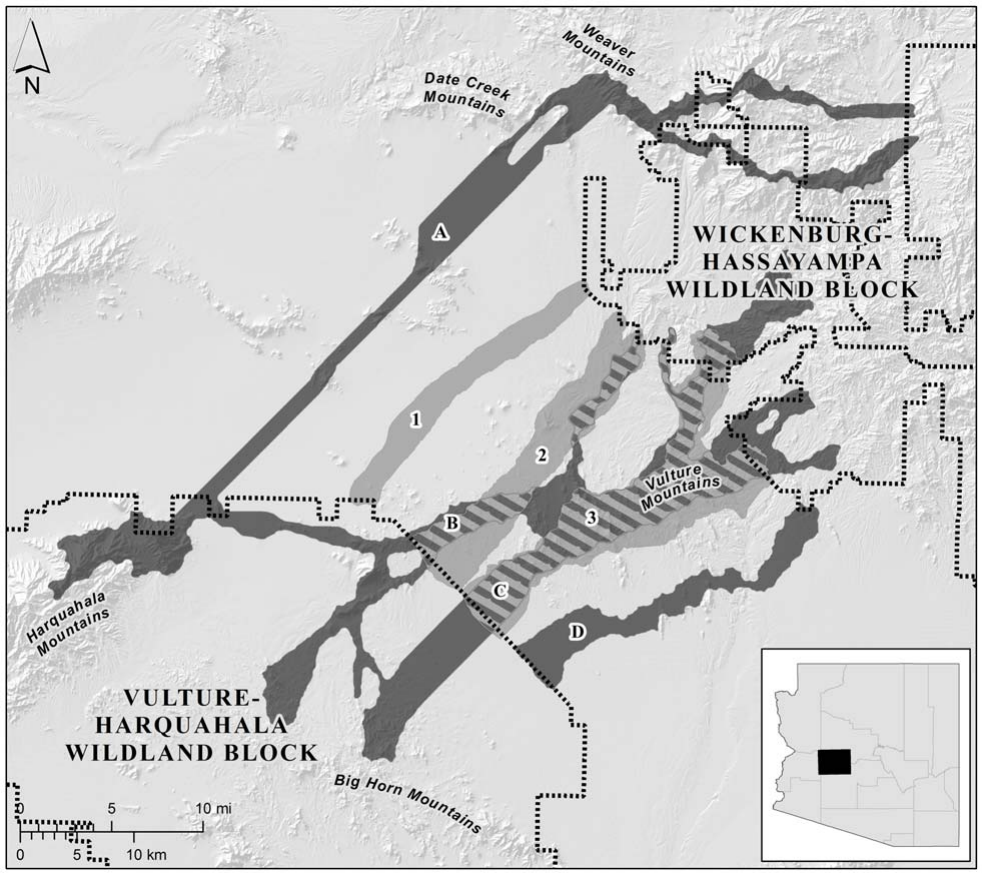
Map of the linkage designs for the Wickenburg-Hassayampa planning area. The land facets design is shown in dark grey and the focal species design is shown in light grey; hatching indicates where the two types of linkage designs overlap. Linkage strands in the land facets design consisted of corridors for (A) high elevation, steep canyon bottoms and ridges; (B–C) low elevation, gentle canyon bottoms and ridges; low elevation, steep canyon bottoms and ridges; mid elevation, steep, cool slopes; high elevation, steep, warm slopes; and high diversity of land facets; and (D) low elevation, gentle, warm slopes. Linkage strands in the focal species design consisted of corridors for (1) badger; (2) black-tailed jackrabbit, javelina, and mule deer; and (3) desert bighorn sheep, desert tortoise, and Gila monster. Inset shows location within Arizona, USA.

**Figure 3:**
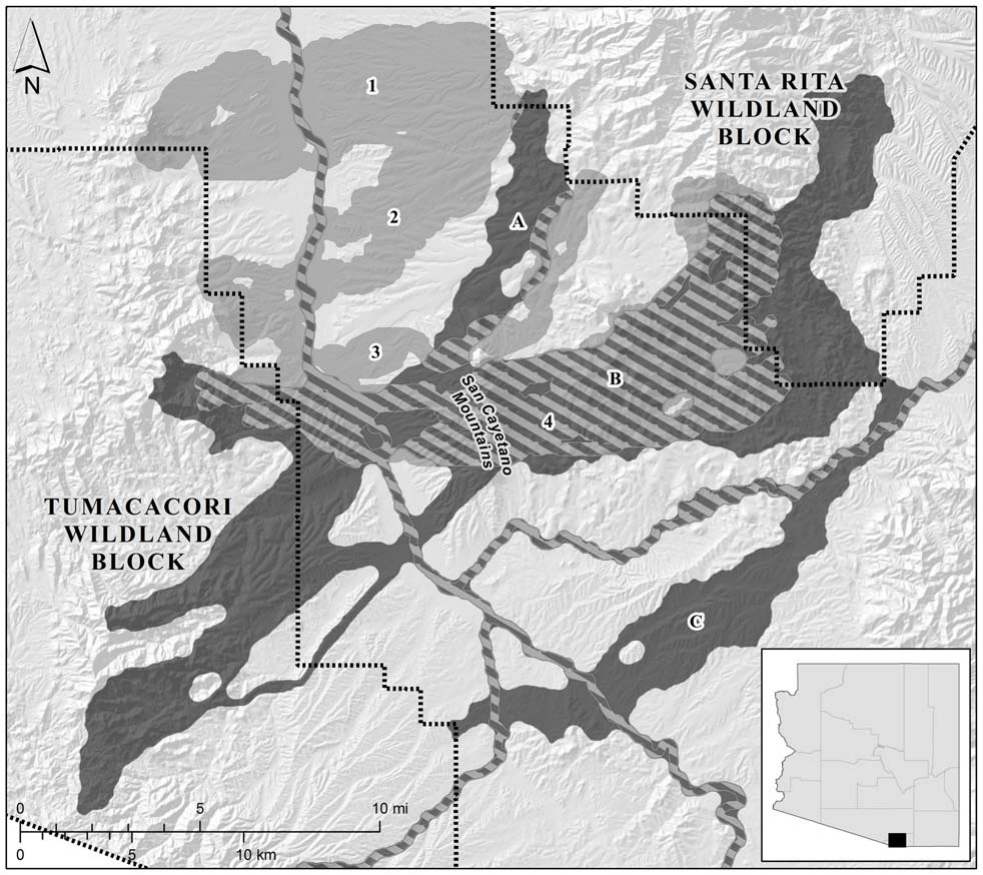
Map of the linkage designs for the Santa Rita-Tumacacori planning area. The land facets design is shown in dark grey and the focal species design is shown in light grey; hatching indicates where the two types of linkage designs overlap. Linkage strands in the land facets design consisted of corridors for (A) high elevation, steep canyon bottoms; (B) low elevation, gentle canyon bottoms and ridges; mid elevation, gentle canyon bottoms and ridges; mid elevation, steep canyon bottoms and ridges; high elevation, steep ridges; mid elevation, steep, cool slopes; mid elevation, steep, hot slopes; high elevation, gentle, hot slopes; and high diversity of land facets; and (C) low elevation, gentle, warm slopes. Linkage strands in the focal species design consisted of corridors for (1) antelope jackrabbit, badger, desert box turtle, jaguar, javelina, mountain lion, mule deer, Sonoran desert toad, and Sonoran whipsnake; (2) Coues' white-tailed deer, porcupine, and white-nosed coati; (3) tiger rattlesnake; and (4) Arizona gray squirrel, black bear, and black-tailed rattlesnake. Inset shows location within Arizona, USA.

We conducted this comparison in three landscapes in Arizona, USA for which linkages have been designed for both focal species and land facets (Table 1; Figures 1, 2, 3). Because true landscape connectivity (for species or land facets) is not known, we examine the performance of one type of design relative to the other. In other words, to evaluate each approach to linkage design, we used as a benchmark the linkage designed under the alternative strategy in the same landscape. We used two metrics to quantify the performance of each linkage design for each focal species, namely resistances along least-cost paths and distances between patches of modeled breeding habitat. Because “breeding patches” cannot be defined for land facets, we used resistances along least-cost paths, and the lengths of the longest high-resistance section of the least-cost path, to quantify how well each linkage design provided continuity of land facets.

**Table 1:** Location, number of focal species and land facets modeled, and size of linkage designs in each of the three planning areas used in our evaluation.

Planning area	Location (centroid)	Size of planning area (km^2^)	Number of focal species modeled	Number of land facets modeled (including the diversity of land facets)	Size of focal species linkage design^1^ (km^2^)	Size of land facets linkage design^1^ (km^2^)	Size of combined linkage designs^1^ (km^2^)	Minimum size of linkage design needed to serve all focal species and land facets^1^^,^ 2 (km^2^)	Previous column as percent of	
									land facets design	focal species design
Black Hills-Munds Mountain	111.9 W, 34.7 N	3817.4	5	12	247.9	431.3	536.2	431.3	100%	174%
Wickenburg-Hassayampa	112.8 W, 33.9 N	9786.6	7	10	340.7	411.8	611.0	411.8	100%	121%
Santa Rita- Tumacacori	111.0 W, 31.6 N	2475.9	16	13	363.3	316.4	473.4	329.0	104%	91%

1 Excluding area inside of the wildland blocks.

2 This design was created by expanding the land facets linkage design so that gaps between breeding patches were no longer than the corresponding gaps in the focal species design. Such expansion was necessary only in the Santa Rita-Tumacacori planning area.

## Methods

### Linkage Planning Areas and Linkage Designs

We selected three areas in Arizona for which linkages have been designed for both land facets and focal species (Table 1; Figures 1, 2, 3). In each planning area, linkages were designed to conserve connectivity between a pair of large, publicly-owned wildlands. Beier et al. [22] describe each area's ecological significance, threats to connectivity, and patterns of land ownership and land cover. Corridors for land facets and focal species were designed using least-cost modeling (i.e., least-cost corridors; [2], [4]), and were based on raster data with a 30-m resolution [9], [22]. Each corridor had an approximate minimum width of 1 km, which was accomplished by increasing the maximum allowable cost of cells included in the corridors until a 1-km width was reached.

Beier et al. [22] describe the procedures used to produce the three linkage designs based on focal species. Each focal species linkage was the union of five to 16 single-species corridors designed to conserve gene flow and demographic stability by supporting dispersal movements of vagile species and multigenerational movement for relatively sedentary species (Table 2; Figures 1, 2, 3) [22]. Focal species were selected for their area sensitivity, barrier sensitivity, and range of vagilities and habitat specificities; no species had a life history that included migrating between wildland blocks. Least-cost corridors for focal species were modeled using the inverse of habitat quality (see *Continuity of Modeled Breeding Patches for Focal Species* below).

**Table 2:** Relative performance of linkage designs with respect to focal species.

Planning area	Locally widespread species for which resistance profiles suggest that the land facets design and focal species design performed equally well	Utility assessed on the basis of distances between modeled breeding patches and resistance profiles		
		Species for which the land facets and focal species designs performed equally well	Species for which the land facets design performed better than the focal species design	Species for which the land facets design performed worse than the focal species design
Black Hills-Munds Mountain	Javelina^1^ (*Tayassu tajacu*)	Mountain lion (*Puma concolor*)	Black bear^2^ (*Ursus americanus*)	
	Mule deer (*Odocoileus hemionus*)		Elk^3^ (*Cervus elephus*)	
Wickenburg-Hassayampa	Badger (*Taxidea taxus*)	Desert bighorn sheep^1^ (*Ovis canadensis nelsoni*)		
	Black-tailed jackrabbit (*Lepus californicus*)			
	Desert tortoise^1^ (*Gopherus agassizii*)			
	Gila monster^1^ (*Heloderma suspectum*)			
	Javelina^1^ (*Tayassu tajacu*)			
	Mule deer (*Odocoileus hemionus*)			
Santa Rita-Tumacacori	Antelope jackrabbit (*Lepus alleni*)	Black bear (*Ursus americanus*)	Coues' white-tailed deer^5^^,^ 6 (*Odocoileus virginianus couesi)*	Arizona gray squirrel^7^ (*Sciurus arizonensis*)
	Badger (*Taxidea taxus*)	White-nosed coati^4^ (*Nasua narica*)	Mountain lion^5^ (*Puma concolor*)	Black-tailed rattlesnake^1^^,^ 7 (*Crotalus molossuss*)
	Desert box turtle (*Terrapene ornate luteola)*		Porcupine^5^^,^ 6 (*Erethizon dorsatum*)	Tiger rattlesnake^1^^,^ 7 (*Crotalus tigris*)
	Jaguar (*Panthera onca*)			
	Javelina^1^ (*Tayassu tajacu*)			
	Mule deer (*Odocoileus hemionus*)			
	Sonoran desert toad^1^ (*Bufo alvarius*)			
	Sonoran whipsnake^1^ (*Masticophis bilineatus*)			

Footnotes in the last 2 columns indicate the metrics that differed most between the two types of linkage designs.

1 For this species-landscape combination, elevation and topographic position (factors in the land facet models) had a combined weight >50% in the focal species model.

2 The single 26-km gap between modeled breeding patches in the focal species design was much worse than the 2 gaps of 8.6 and 1.9 km in the land facets design (Table 3).

3 The resistance profile was much lower in the land facets design (Figure 5A).

4 The maximum distance between breeding patches was 23% shorter in the land facets design (Table 3), but this was offset by the greater combined length of the two gaps and their higher resistance profiles in the land facets design.

5 Lengths of largest gaps between modeled breeding patches were much shorter in the land facets design (Table 3).

6 Resistance profiles were lower in the land facets design than in the focal species design.

7 Lengths of largest gaps between modeled breeding patches were shorter in the focal species design (Table 3).

Brost and Beier [9] describe the procedures used to produce the three linkage designs based on land facets. Each land facets design was the union of nine to 12 corridors for individual land facets and one corridor with high interspersion of facets. Although Beier and Brost [8] and Brost and Beier [9] recommend defining land facets based on both topographic and soil attributes, soils information in these landscapes was inadequate. Therefore, we defined land facets on the basis of three continuous variables, namely elevation, slope angle, and solar insolation, and one categorical variable, topographic position. In each landscape, most correlations between continuous variables were modest (mean |*r*| = 0.34, range 0.02–0.62). Procedures used to delineate termini (start and end points of corridors) and assign resistance values to cells are summarized briefly below (see *Continuity of Land Facets*) and described in detail by Brost and Beier [9].

### Continuity of Modeled Breeding Patches for Focal Species

Patches of habitat large enough to support breeding by a species can serve as stepping stones within a linkage, reducing the amount of unsuitable habitat the species must cross in a single event while moving between wildland blocks. Our two metrics of habitat continuity relied on modeled breeding patches. Beier et al. [22] identified breeding patches for each focal species by joining adjacent cells of modeled breeding habitat into clusters that exceeded the species' average home range size. Habitat quality values, estimated from scientific literature and expert opinion, were assigned to levels of each of 4 habitat factors (land cover, elevation, topographic position, and distance to paved road); individual factors were combined into an overall index using a weighted geometric mean. Values ranged between 1 (best) and 10 (worst), and were assigned relative to a value of 5 that marked the threshold between breeding and non-breeding habitat. Although other procedures provide marginally more accurate habitat patches in some situations [23], these simple patches provide a convenient way to compare linkage designs.

**Figure 4:**
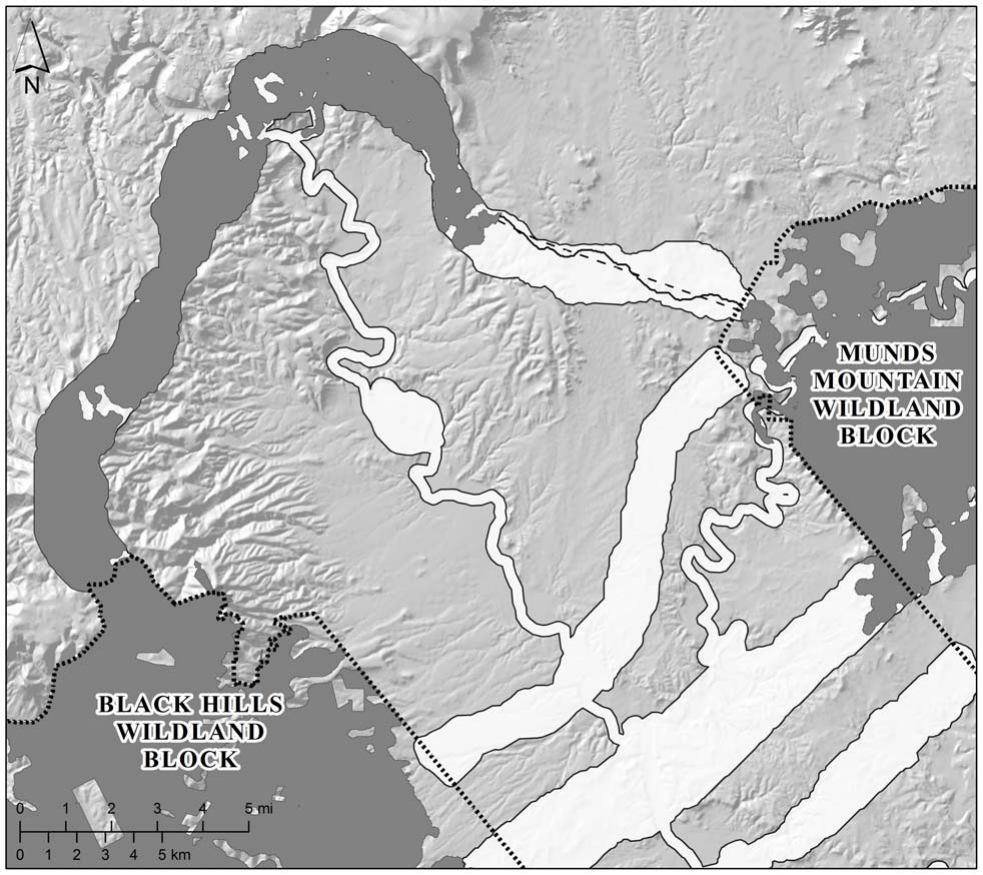
Metrics used to evaluate the performance of linkage designs with respect to focal species. This illustration shows the distance between breeding patches (dashed line) and the associated least-cost path (solid line) for elk in the Black Hills-Munds Mountain planning area under the focal species linkage design (swaths of land between wildland blocks). Elk breeding patches within wildland blocks or the linkage design are indicated by dark grey. The resistance profile corresponding to the least-cost path is a graph of the resistance of each cell in the least-cost path plotted against distance along that path (see Figure 5A). Distance measurements and least-cost paths were constrained to pass through areas contained by the linkage design. Note that the western terminus of the elk corridor (the breeding patch in the Black Hills Wildland Block) is contiguous with a breeding patch in the focal species linkage design, resulting in a single, relatively short, interpatch gap. For comparison, both metrics were also calculated using breeding patches contained in the land facets linkage design (not illustrated).

Our first metric of continuity of breeding habitat was a list of the Euclidean distances between modeled breeding patches for each species within each type of linkage design (Figure 4); these reflect gaps between stepping stones of breeding habitat. Corridor termini (breeding patches wholly contained within the wildland blocks) served as starting/ending points for the measurements, which were made through the strand of the linkage that minimized the longest distance between patches.

**Figure 5:**
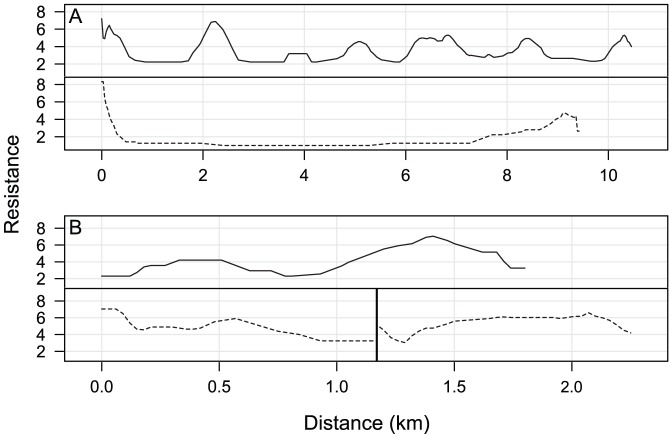
Example resistance profiles between modeled breeding patches for focal species. The paired profiles in this figure depict species-specific resistance in the focal species (solid line) and land facets (dashed line) linkage designs. (A) Elk in the Black Hills-Munds Mountain planning area. Although the gap between breeding patches was approximately the same in both designs (and thus the length of the resistance profiles are approximately the same), the resistance profile from the land facets design contains lower resistance values. (B) White-nosed coati in the Santa Rita-Tumacacori planning area. The relatively long gap between breeding patches in the focal species design was offset by the collective length of the two gaps in the land facets design and their slightly higher resistance profiles. The vertical line indicates a breeding patch between two gaps; line width does not indicate the width of the breeding patch. To smooth distracting peaks and troughs in resistance values, these profiles show the running mean resistance of the cell and its seven previous and seven succeeding cells, with smaller moving averages for the first and last seven resistance values in the profile. See Supporting Information for the raw, unsmoothed resistance profiles and smoothed profiles for all species.

Our second metric was the resistance profile of the least-cost path connecting consecutive patches (Figure 4). A resistance profile is a graph of the species-specific resistance of each cell in the least-cost path plotted against distance along that path (e.g., Figure 5). Least-cost paths are similar to least-cost corridors in that both minimize cumulative resistance across the matrix; however, a path is only 1 cell (30 m) wide. We generated a resistance profile for each gap between patches of modeled breeding habitat for focal species in each landscape. We used the Spatial Analyst extension of ArcGIS 9.3 to identify least-cost paths.

For 18 of the 28 species-landscape combinations, we assessed continuity of breeding patches solely on the basis of resistance profiles between corridor termini. These included 16 cases of species with “locally widespread” habitat, meaning that ≥90% of the matrix between the wildland blocks consisted of modeled breeding habitat for that species. For these species, either the entire area was one breeding patch and interpatch distance was not defined, or only a single, short (i.e., <100 m) gap between breeding patches existed. In another case (mountain lion in the Black Hills-Munds Mountain planning area), although the species did not have locally widespread habitat, no gaps between breeding patches existed under either design. In the last case (desert bighorn sheep in the Wickenburg-Hassayampa area), modeled breeding patches occurred only in the wildland blocks, such that the entire matrix was a single gap.

When paired resistance profiles (i.e., the profiles for one species and landscape in both types of linkage designs) differed in length but were similar in resistance, the shorter profile was considered better. When the paired profiles were approximately equal in length, we considered the two profiles equivalent if both had resistance values <5 (i.e., sufficient habitat quality, although not necessarily sufficient area, for breeding) along their entire length, or if both contained similar values (including values>5) along their entire length. We considered the difference between profiles to be biologically significant if one profile was predominantly below 5 resistance units while the other was predominantly above 5 units. In the few cases when length and resistance differed in more complex ways, we evaluated interactions between length and resistance (relative to the threshold value of 5) in light of a species' average dispersal distance.

We considered and rejected two other simple metrics that could be used to compare linkage designs, namely percent overlap of the linkages and mean resistance of the profiles, and another metric based on electrical circuit theory, namely resistance distance between patches [24]. Low values of percent overlap are not meaningful because two non-overlapping linkages often provide similar connectivity [25]. Mean resistance can yield spurious results because it does not reflect the spatial distribution of habitat quality. For example, a profile with low resistance (4 resistance units) over 90% of its length and a complete barrier (10 units) over the remaining 10% has the same mean resistance (4.6) as a superior profile in which all cells had a uniform resistance of 4.6 units. For another example, a profile 5 km long with a uniform resistance of 4 units has a *higher* mean resistance (4.0) than an *inferior* profile 10 km long with a resistance of 4 units for 5 km and resistance of 3.8 units for the remaining 5 km (mean 3.9). Because we could compare profiles to the threshold value of 5, we did not have to use mean resistance to compare designs. Although resistance distance has the advantage of incorporating the contributions that multiple dispersal pathways make to connectivity, its main disadvantage is that it lacks biological interpretation because it does not preserve the original scale upon which resistance for species was defined. The mean resistance distance also fails to reflect the spatial arrangement of permeable habitat, and is thus subject to the same shortcomings as mean resistance noted above. Although a pixel-wide path surrounded by inhospitable matrix would poorly represent resistance, we never found this to be the case and we believe that resistance profiles meaningfully represent the resistance an animal would encounter moving between patches.

### Continuity of Land Facets

We generated resistance profiles between corridor termini to evaluate how well each type of linkage design provided connectivity for each land facet. Here, a profile is a plot of land-facet resistance against distance along the least-cost path. Corridor termini were the largest polygons within the wildland blocks dominated by the focal land facet [9]. Resistance for land facets was measured using Mahalanobis distance, a multivariate measure of dissimilarity [26] of each cell from an ‘ideal’ or characteristic elevation, slope angle, solar insolation, and relative density of the focal land facet within a circular neighborhood with a 3-cell radius [9]. Resistance values for land facets reflect the departure of a cell from the prototypical cell of the focal facet type, and are measured in multivariate standardized units (analogous to a standard deviation in a univariate analysis). These resistance values have a minimum of 0 (which occurs when a cell has the mean characteristics of that land facet), and no theoretical maximum.

We also generated resistance profiles to evaluate how well each type of linkage design provided connectivity for diversity of land facets. In this case, corridor termini were the largest polygons consisting of the most diverse cells inside the wildland blocks (for details see [9]). The complement of Shannon's Evenness (*E_H_*) [27] was used to measure richness and evenness of land facets within a circular neighborhood with a 5-cell radius:
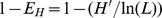
where *L* is the number of land facets in a particular landscape, H' is Shannon's index, and ln(*L*) is the maximum value of Shannon's index. These values are scaled [0, 1], where 0 occurs when all land facets occur in equal proportions, and 1 occurs when the neighborhood contains only 1 land facet type. These values were used as a resistance surface in least-cost modeling [9], thus producing a corridor that optimized connectivity for high land facet diversity.

The interpretation of resistance values for land facets is less clear than resistance values for focal species because we do not know how Mahalanobis distance or Shannon's Evenness relates to resistance to movement of species associated with land facets. Therefore, the resistance profiles can suggest which linkage design performed better, but the biological significance of a difference is not clear.

We used two metrics to compare land facet connectivity between the two types of linkage designs. First we compared the mean resistances of the two resistance profiles. Because the resistance scale for land facets lacked a meaningful reference value (such as the threshold of 5 resistance units for focal species), we used mean resistance despite difficulties in interpreting it (see *Continuity of Modeled Breeding Patches for Focal Species*). To mitigate these difficulties, we examined profiles for artifacts that could give rise to spurious inferences. Second, we measured the length of the longest high-resistance segment in each land facet profile. To identify high-resistance segments, we rescaled Mahalanobis distances to [0, 1] by calculating the p-value associated with each Mahalanobis distance [26], and then identified the longest segment of continuous p-values <0.05. Under multivariate normality, Mahalanobis distances are approximately χ^2^ distributed [26]. Because our data were not multivariate normal, the p-values do not indicate statistical significance. Nonetheless, p-values closer to 0 indicate enormous dissimilarity between a cell and the focal facet type, and the longest segment of p-values <0.05 is a consistent metric to compare resistance profiles. Both metrics were calculated using the raw, unsmoothed resistance values.

We assessed the relative performance of the two linkage designs by examining the differences between metrics for paired resistance profiles. A lower mean resistance, or shorter high-resistance segment, indicates superior, but not necessarily biologically better, performance.

## Results

Each focal species linkage design included five to 16 individual species corridors, which overlapped to produce three to four major strands per linkage (Table 1, Figures 1, 2, 3). Each land facets design contained 10–13 land facet corridors, which overlapped to produce three to five strands per linkage. Compared to the linkages designed for focal species, the linkage designed for land facets was 21% larger in the Wickenburg-Hassayampa planning area, 74% larger in the Black Hills-Munds Mountain planning area, and 15% smaller in the Santa Rita-Tumacacori planning area (Table 1; Figures 1, 2, 3).

### How Well Did Each Linkage Design Provide Continuity of Breeding Patches?

Of the 28 species-landscape combinations, 16 focal species had locally widespread habitat (Table 2). For these species, resistance profiles did not differ substantially between the focal species and land facets linkage designs (See Supporting Information). The largest apparent difference was for badger in the Wickenburg-Hassayampa and Santa Rita-Tumacacori planning areas, for which values in the resistance profiles were about 2 units greater (on a scale of 1–10) under the land facets designs than under the focal species designs.

Of the 12 remaining species-landscape combinations, four had similar continuity of modeled breeding patches in both designs, five species had greater continuity in the land facets linkage design, and 3 species had more connected breeding patches in the focal species design (Tables 2 and 3). We did not expect land facets designs to provide better connectivity in the five species-landscape situations; maps of modeled breeding patches helped elucidate these cases. For example, breeding patches for black bear and elk in the Black Hills-Munds Mountain planning area were most continuous in the linkage strand for high elevation land facets (Figure 1, strand A), which corresponded to the forest types associated with these species [22]. The three cases in which profiles of species-specific resistance were lower in the land facets designs than in the focal species designs were even more surprising (e.g., Figure 5A). In all three cases, the land facets design provided a longer corridor for the focal species, but with shorter distances between breeding patches and lower inter-patch resistances than the focal species design. In one of these three cases, the best land facet strand for elk (Figure 1, strand A) was much longer than the “elk corridor” (Figure 1, strand 1).

**Table 3:** Distances between breeding patches for focal species.

Planning area	Focal species	Distances between breeding patches under focal species linkage design (km)	Distances between breeding patches under land facets linkage design (km)
Black Hills-Munds Mountain	Black bear	26.01	8.55
			1.87
	Elk	9.13	8.66
Wickenburg-Hassayampa	Desert bighorn sheep	41.89	42.65
Santa Rita-Tumacacori	Antelope jackrabbit	0.03	0.03
	Arizona gray squirrel^1^	3.37	4.53
		3.21	2.11
		0.98	1.55
		0.90	0.98
		0.90	0.90
		0.86	0.90
	Black-tailed rattlesnake	1.39	1.39
		0.72	1.24
		0.23	1.17
		0.16	0.72
		0.07	0.07
		0.06	
	Black bear	7.87	7.87
	Coues' white-tailed deer	1.45	0.69
			0.67
	Jaguar	0.11	0.19
	Mountain lion	3.70	1.70
	Porcupine	0.63	0.30
		0.03	0.19
	Tiger rattlesnake	0.47	1.38
		0.46	0.73
		0.39	0.70
		0.18	0.11
		0.11	0.03
	White-nosed coati	1.33	1.02
			0.99

Only species with gaps between modeled breeding patches are listed. Except for Arizona gray squirrel in the Santa Rita-Tumacacori planning area, distances between all breeding patches are listed (see footnote).

1 Arizona gray squirrel had 22 gaps between breeding patches under both types of linkage designs (mean length of gaps: focal species design = 0.70 km; land facets design = 0.74 km). No other species had >6 gaps in any landscape.

### How Well Did Each Linkage Design Provide Continuity of Land Facets?

**Figure 6:**
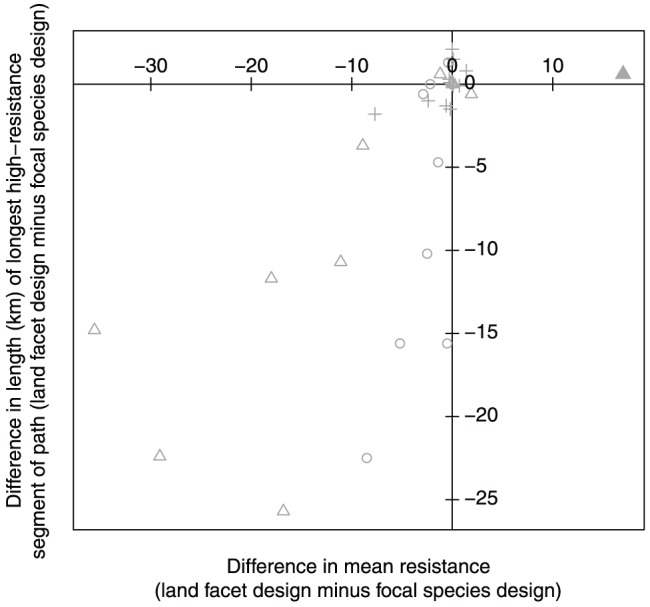
Differences between paired resistance profiles for 32 land facet-landscape combinations. Differences were calculated as the land facet value minus the focal species value. Points in the lower left quadrant (negative x- and y-values) represent cases in which the profile in the focal species design had higher mean resistance and a longer high-resistance segment than the corresponding profile in the land facets design. Each planning area is indicated by a different symbol: Black Hills-Munds Mountain (▵), Wickenburg-Hassayampa (°), and Santa Rita-Tumacacori (+). The solid triangle corresponds to the footnote in Table 4.

**Figure 7:**
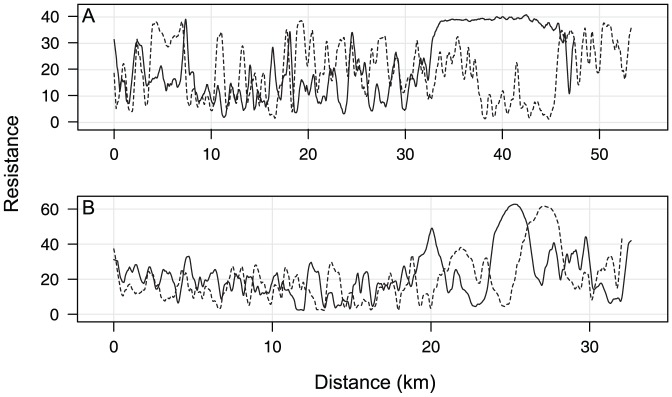
Example resistance profiles between corridor termini for land facets. The paired profiles in this figure depict land-facet resistance in the focal species (solid line) and land facets (dashed line) linkage designs. (A) Mid elevation, steep, cool slopes in the Wickenburg-Hassayampa planning area. Although these profiles have approximately the same mean resistance, the profile in the focal species design has a longer high-resistance segment (portion of profile between ∼30–46 km) than the profile in the land facets design (portion of profile between ∼26–32 km). (A) Low elevation, gentle ridges in the Santa Rita-Tumacacori planning area. These profiles have approximately the same mean resistance and length of high-resistance segment. To smooth distracting peaks and troughs in resistance values, these profiles show the running mean resistance of the cell and its seven previous and seven succeeding cells, with smaller moving averages for the first and last seven resistance values in the profile. See Supporting Information for the raw, unsmoothed resistance profiles and smoothed profiles for all land facets.

Twelve of the 32 land facet-landscape combinations had substantially higher mean resistance and longer high-resistance segments in the focal species linkages than in the land facets linkages, including six of 11 land facets in the Black Hills-Munds Mountain planning area, five of nine land facets in the Wickenburg-Hassayampa planning area, and one of 12 facets in the Santa Rita-Tumacacori area (Figures 6 and 7; Table 4). Mean resistance of profiles for the high diversity of land facets was trivially higher (0.05–0.1 resistance units on a scale of 0–1) in the focal species design in all three planning areas (Table 4). There was only one case in which a focal species linkage provided lower mean land-facet resistance than the land facets linkage (solid triangle in Figure 6), but the full resistance profile (See Figure S6: 9^th^ panel) demonstrated that the two designs provided similar connectivity (Table 4 footnote).

**Table 4:** Mean resistance and longest high-resistance segments of resistance profiles for land facets under the two types of linkage designs.

		Mean resistance		Longest segment of resistance profile with p-values <0.05 (km)	
Planning area	Land facet	land facets design	focal species design	land facets design	focal species design
Black Hills-Munds Mountain	Canyon bottoms: low elevation, gentle	19.5	30.6	6.4	17.1
	Canyon bottoms: mid elevation, steep	33.0	33.0	27.9	27.8
	Canyon bottoms: high elevation, gentle	21.2	38.0	16.0	41.7
	Ridges: low elevation, gentle	14.6	32.6	5.6	17.3
	Ridges: mid elevation, steep	40.3	40.3	28.9	28.9
	Ridges: high elevation, gentle	17.1	46.2	14.4	36.8
	Slopes: low elevation, gentle, warm	3.1	12.0	0.4	4.1
	Slopes: low elevation, steep, cool	26.0	27.2	16.6	16.0
	Slopes: mid elevation, gentle, warm^1^	56.4	39.4	26.5	25.9
	Slopes: mid elevation, steep, warm	33.5	31.6	20.4	21.0
	Slopes: high elevation, gentle, hot	22.5	58.1	26.5	41.3
	High diversity	0.43	0.48	---	---
Wickenburg-Hassayampa	Canyon bottoms: low elevation, gentle	29.8	29.8	18.7	18.7
	Canyon bottoms: low elevation, steep	29.5	30.0	48.4	64.0
	Canyon bottoms: high elevation, steep	32.9	38.1	63.1	78.7
	Ridges: low elevation, gentle	30.1	30.5	21.8	20.5
	Ridges: low elevation, steep	30.1	31.5	18.1	22.8
	Ridges: high elevation, steep	28.1	36.6	50.0	72.5
	Slopes: low elevation, gentle, warm	0.6	3.5	0.0	0.6
	Slopes: mid elevation, steep, cool	19.0	21.5	6.3	16.5
	Slopes: high elevation, steep, hot	14.1	16.3	6.6	6.6
	High diversity	0.45	0.55	---	---
Santa Rita-Tumacacori	Canyon bottoms: low elevation, gentle	25.1	25.1	9.5	7.4
	Canyon bottoms: mid elevation, gentle	38.4	38.6	20.8	20.7
	Canyon bottoms: mid elevation, steep	26.0	26.0	9.2	9.2
	Canyon bottoms: high elevation, steep	47.2	47.8	19.8	21.1
	Ridges: low elevation, gentle	20.0	22.4	6.8	7.8
	Ridges: mid elevation, gentle	33.3	33.6	18.0	17.7
	Ridges: mid elevation, steep	25.9	25.2	8.9	9.0
	Ridges: high elevation, steep	49.4	49.6	18.7	20.2
	Slopes: low elevation, gentle, warm	2.3	10.0	0.2	2.0
	Slopes: mid elevation, steep, cool	31.3	29.9	12.3	11.5
	Slopes: mid elevation, steep, hot	29.1	29.0	9.6	9.7
	Slopes: high elevation, gentle, hot	25.9	25.8	32.6	31.1
	High diversity	0.47	0.47	---	---

Resistance was calculated as Mahalanobis distance (minimum 0, no theoretical maximum) for land facets, and as the complement of Shannon's evenness (0 to 1) for land facet diversity.

1 Although the mean resistance in the focal species design was lower than in the land facets design, the resistance profiles were nearly identical except for an additional 7 km segment of low resistance in the focal species profile that reduced its mean value. This was the only pair of profiles where a profile pattern counteracted a large difference in mean resistance.

## Discussion

### Do Linkages Designed for Land Facets Provide Continuity of Modeled Breeding Patches?

For 25 of 28 focal species-landscape combinations, linkages designed for land facets connected patches of modeled breeding habitat as well as or better than focal species designs (Table 2). For the 16 species-landscape combinations with locally widespread habitat, similar performance under both types of designs was inevitable given the distribution of these species' habitat. In fact, any linkage design that excluded urban or disturbed areas would likely have performed well for these species.

The other 12 species-landscape combinations, in which the focal species had patchily distributed habitat, provide a more meaningful assessment of the land facets approach to linkage design. Among these species, the land facets designs performed as well as or better than the focal species designs for all large mammals, and for two medium-sized mammals, namely porcupine and white-nosed coati (Table 2). The land facets design performed worse for Arizona gray squirrel, black-tailed rattlesnake, and tiger rattlesnake in one planning area. Breeding patches for these three species were also the most narrowly distributed of all species-landscape combinations we studied, suggesting that species with limited habitat in the planning area tend to be better served by a focal species approach.

Shorter distances or lower resistances for species between breeding patches do not necessarily translate into increased connectivity, which ultimately depends on the interaction between the linkage design and species traits such as mobility, behavior, and generation time. For example, shorter distances between breeding patches under the land facets design for Coues' white-tailed deer and mountain lion in the Santa Rita-Tumacacori planning area (Table 3) might not indicate substantially better connectivity for these highly mobile species [28], [29]. In contrast, small increases (0.9–1.2 km) in inter-patch gaps can be important for less-mobile species like Arizona gray squirrel, black-tailed rattlesnake, and tiger rattlesnake. Similarly, substantially shorter distances between breeding patches for black bear and lower resistance between patches for elk under the land facets design in the Black Hills-Munds Mountain planning area are almost certainly biologically meaningful for these species (Table 3; Figure 5A).

Because least-cost analysis minimizes resistance-weighted distance between termini rather than Euclidean distance between patches, we were not surprised to find that the land facets designs sometimes provided shorter distances between breeding patches than the focal species designs. However, we were surprised that for some species the land facets designs simultaneously provided shorter distances between patches and profiles of species resistance similar to or lower than those of the focal species designs (e.g., black bear and elk in the Black Hills-Munds Mountain planning area; Table 2). For these species, the land facet strand that optimized continuity of breeding patches was relatively long but biologically more effective because breeding patches occupied most of the length of the strand. Beier et al. [2] suggested that least-cost models for focal species could be modified to produce corridors that minimize inter-patch gaps (rather than cumulative resistance). Specifically, they suggested that assigning near-zero resistance to breeding patches would result in longer corridors dominated by breeding patches, with shorter inter-patch gaps. Although Beier et al. [2] recommended this procedure only for species requiring multiple generations to traverse a corridor, we suggest this modification might be useful for more mobile species as well. We recommend analysts make this choice by carefully considering how a particular species in a particular landscape perceives the difference between breeding habitat and less hospitable matrix, and how the species makes gap-crossing decisions.

The use of habitat quality as a surrogate for resistance to movement in least-cost models for focal species implies that species select dispersal routes (between termini and between breeding patches) in the same way they select habitat. Although this assumption is reasonable for species that require multiple generations to move between wildland blocks and thus need places to live and reproduce en route, more mobile species can disperse through poor quality habitat or habitats not used for other life history needs (e.g., [30]). For these species, models based on habitat quality may underestimate connectivity. More rigorous estimates of resistance could be derived from data on animal movement or genetic patterns; however, expert opinion and literature review are often used because of time and budgetary constraints [2].

### Do Linkages Designed for Focal Species Provide Continuity of Land Facets?

For 20 of 32 land facet-landscape combinations, differences between resistance profiles in focal species and land facets linkages were minor (Figure 6; Table 4). Profiles for the other 12 land facets had substantially higher mean land-facet resistance and longer high-resistance segments in the focal species linkages.

Focal species linkages probably performed well for some land facets because a focal species was associated with that facet type. For example, the focal species design in the Wickenburg-Hassayampa planning area provided good continuity for low elevation canyon bottoms and ridges because these features were important factors in the habitat models [22] for desert bighorn sheep (ridges) and Gila monster (ridges and canyon bottoms). In contrast, no focal species in this landscape was closely associated with mid-elevation, steep, low-insolation slopes, so the focal species design provided little connectivity for this facet type.

In landscapes where topographically diverse terrain in the matrix is restricted to a small area between wildland blocks, a focal species linkage design using diverse focal species will likely provide good continuity for many land facets. In the Santa Rita-Tumacacori planning area, for example, the rugged San Cayetano Mountains lie between the wildland blocks. Because these provided the only mountainous terrain in the matrix, 11 of 12 land facet corridors and the high diversity corridor (Figure 3, strands A and B) passed through the San Cayetanos. So did corridors for Arizona gray squirrel, black bear, black-tailed rattlesnake, and tiger rattlesnake (Figure 3, strands 3 and 4), resulting in extensive overlap—and similar performance—between the two types of linkage designs.

### Implications for Linkage Design

Our results are consistent with the underlying principle of the land facets approach to linkage design, which is that diverse physical environments support diverse biota. Although our example defined facets solely on the basis of topographic variables, data on soils or surficial geology should be used to help define land facets in planning areas where such data are available [7].

In areas where a focal species approach to linkage design is not possible, our results suggest that conservation practitioners may be able to implement a land facets approach with some confidence that the linkage design would serve most potential focal species. However, linkages designed for land facets will perform poorly for some species, such as those with limited habitat in the planning area. Conversely, focal species designs provided less continuity for nearly a third of land facets. Therefore focal species linkage designs by themselves do not reliably provide connectivity for land facets, and thus might not provide connectivity under future climate regimes.

The land facets approach to linkage design should complement, rather than replace, focal species approaches [8]. But simply combining the two types of linkage designs would produce a very large linkage design that would be expensive to conserve. For example, combining designs in our three landscapes could result in a linkage design 30% to 116% larger than the focal species design (Table 1). Because land facets designs provide good connectivity for most focal species, such a simple union of linkage designs would be needlessly large. Indeed, in two of our areas, the land facets design performed as well as or better than the focal species design for all land facets and focal species. In the third landscape, a conservation planner could efficiently provide connectivity for all species and facets by expanding the land facets design to encompass some of the same breeding patches for Arizona gray squirrel, black-tailed rattlesnake, and tiger rattlesnake that are contained in the focal species design. The new design would only be 4% larger than the original land facets design and 9% smaller than the original focal species design (Table 1).

The biggest limitation of our evaluation is that it compared these approaches in only three landscapes, precluding inferences about how much the size of a linkage design affects the utility of the design. Quite likely, the land facets linkage designs provided connectivity for focal species in part due to long, looping corridors for some land facets, resulting in land facet linkage designs that were larger than the focal species designs in two of three cases (Table 1; Figures 1 and 2). To better understand the relationships between linkage area and linkage performance, future work could compare various linkages to randomly-generated linkage designs, or design linkages for many landscapes and statistically adjust for area.

Although the three landscapes we selected were topographically diverse, and the evaluations involved diverse species, additional evaluation is necessary to determine whether land facet linkage designs work well in other landscapes. Evaluations conducted in landscapes where many focal species have narrowly distributed habitat would be particularly informative. Additional analyses could also help develop a general strategy for using land facets in linkage design by suggesting the minimum width of linkage strands and how best to combine linkages based on land facets and focal species. The locations of modeled corridors depend on what variables are used to define land facets and species-specific habitat quality, as well as the structure of those models. The effect of these modeling choices on metrics of linkage performance deserves further attention.

All of our linkage designs were large, and therefore costly to acquire or manage for conservation. They also contained multiple strands with long edges, making them difficult to manage. Thus it would be helpful to develop procedures to modify the linkage design to minimize total area and edge while maintaining connectivity for species or land facets. Such procedures would also help decision-makers evaluate the ecological value of alternative corridor designs proposed as compromises.

We developed the land facets approach to help conservation planners design linkages that will be robust to climate change. We consider land facets to be conceptually the same as the “ecological land units” of Anderson and Ferree [7], who demonstrated that many species of plants and animals are closely linked to these units. We encourage using various types of abiotic land units, as well as other climate-robust connectivity concepts, to design linkages for climate change. As such designs are developed, procedures similar to those in this paper can evaluate how well each design meets the goals of alternative designs, and how outputs of the various designs can best be merged while limiting total area and edge of the design.

## Supporting Information

Figure S1 **Resistance profiles for species with locally widespread habitat and mountain lion in the Black Hills-Munds Mountain planning area.** The smoothed resistance profiles (in bold) are superimposed on the raw, unsmoothed profiles (thinner, fainter lines).
(PDF)

Figure S2 **Resistance profiles for species with locally widespread habitat and desert bighorn sheep in the Wickenburg-Hassayampa planning area.** The smoothed resistance profiles (in bold) are superimposed on the raw, unsmoothed profiles (thinner, fainter lines).
(PDF)

Figure S3 **Resistance profiles for species with locally widespread habitat in the Santa Rita-Tumacacori planning area.** The smoothed resistance profiles (in bold) are superimposed on the raw, unsmoothed profiles (thinner, fainter lines).
(PDF)

Figure S4 **Resistance profiles corresponding to the gaps between breeding patches for black bear and elk in the Black Hills-Munds Mountain planning area.** Each vertical line indicates a breeding patch between two gaps; line width does not indicate the width of the breeding patch. The smoothed resistance profiles (in bold) are superimposed on the raw, unsmoothed profiles (thinner, fainter lines).
(PDF)

Figure S5 **Resistance profiles corresponding to the gaps between breeding patches for focal species in the Santa Rita-Tumacacori planning area.** Each vertical line indicates a breeding patch between two gaps; line width does not indicate the width of the breeding patch. The smoothed resistance profiles (in bold) are superimposed on the raw, unsmoothed profiles (thinner, fainter lines).
(PDF)

Figure S6 **Resistance profiles for land facets in the Black Hills-Munds Mountain planning area.** The smoothed resistance profiles (in bold) are superimposed on the raw, unsmoothed profiles (thinner, fainter lines).
(PDF)

Figure S7 **Resistance profiles for land facets in the Wickenburg-Hassayampa planning area.** The smoothed resistance profiles (in bold) are superimposed on the raw, unsmoothed profiles (thinner, fainter lines).
(PDF)

Figure S8 **Resistance profiles for land facets in the Santa Rita-Tumacacori planning area.** The smoothed resistance profiles (in bold) are superimposed on the raw, unsmoothed profiles (thinner, fainter lines).
(PDF)

Figure S9 **Resistance profiles for high diversity of land facets in the Black Hills-Munds Mountain, Wickenburg-Hassayampa, and Santa Rita-Tumacacori planning areas.** The values in the profiles are the compliment of Shannon's evenness, where 0 is the lowest possible resistance and 1 is the maximum deviation from the optimal Shannon's index value. The smoothed resistance profiles (in bold) are superimposed on the raw, unsmoothed profiles (thinner, fainter lines).
(PDF)
