# Supplementary figures and images for: Comparing Linkage Designs Based on Land Facets to Linkage Designs Based on Focal Species
Source: PLoS One. 2012 Nov 12;7(11):e48965. doi: 10.1371/journal.pone.0048965 (PMC3495916; doi:10.1371/journal.pone.0048965)

Design Type  
Focal species — Land facets

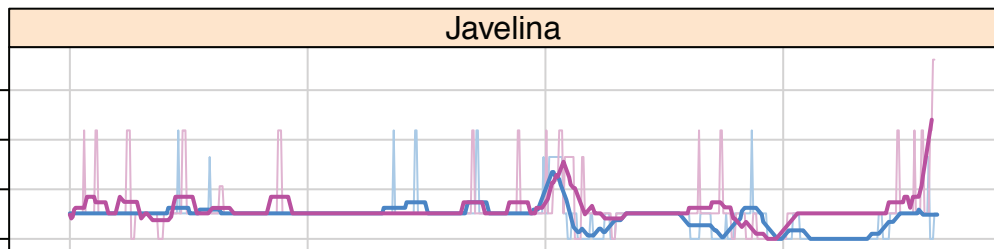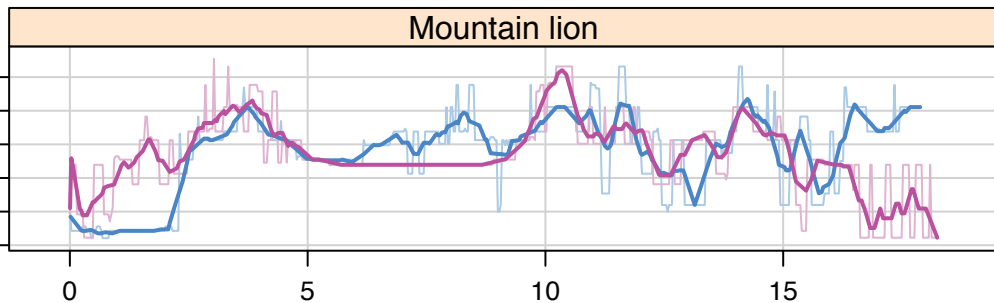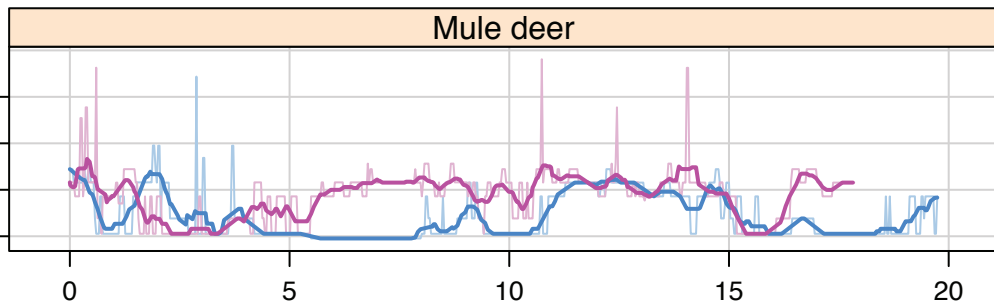

Distance (km)

Supplement: Figure S1 — Resistance profiles for species with locally widespread habitat and mountain lion in the Black Hills-Munds Mountain planning area. The smoothed resistance profiles (in bold) are superimposed on the raw, unsmoothed profiles (thinner, fainter lines). (PDF) [file pone.0048965.s001.pdf]

Design Type  
Focal species — Land facets

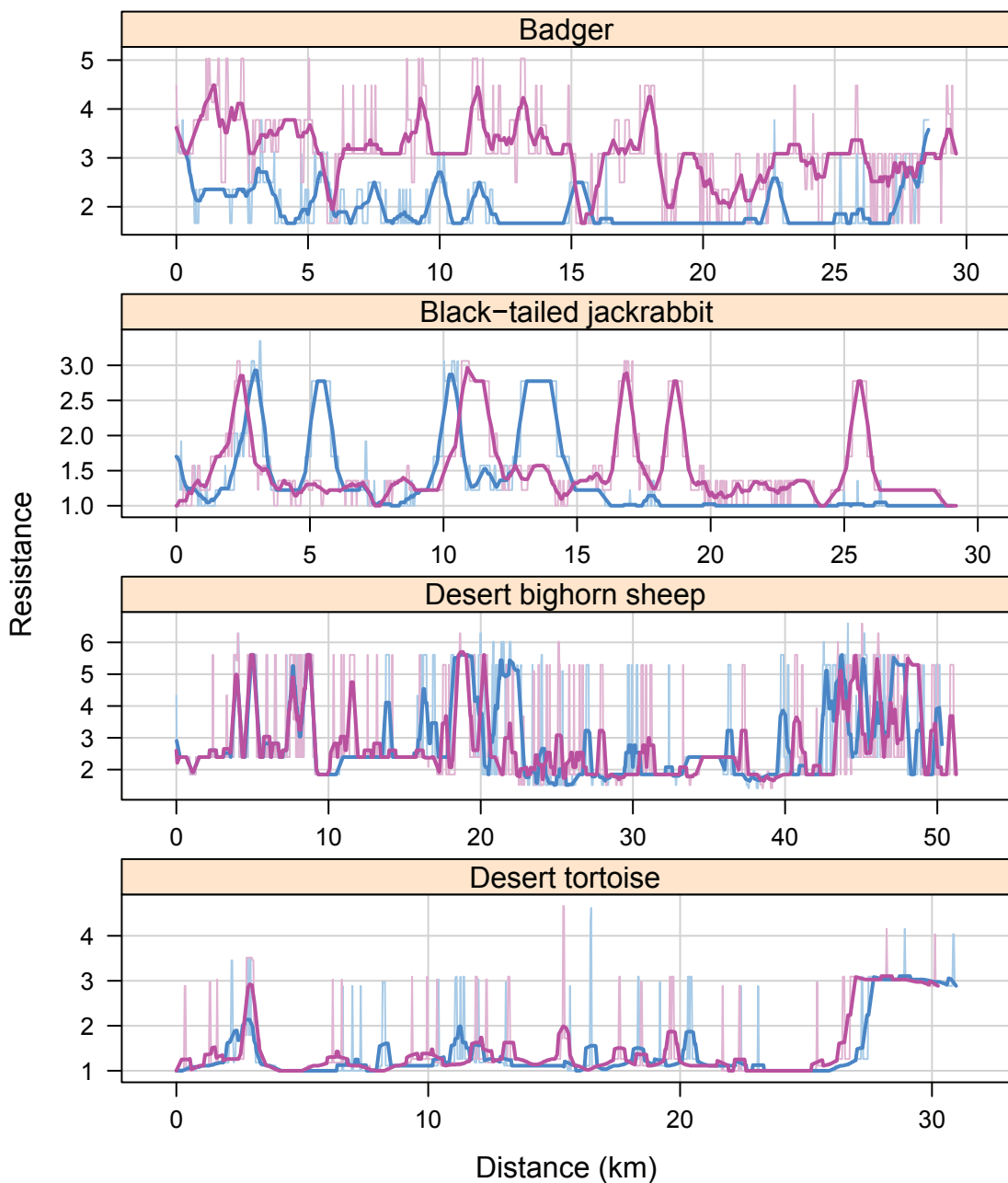

Design Type  
Focal species — Land facets

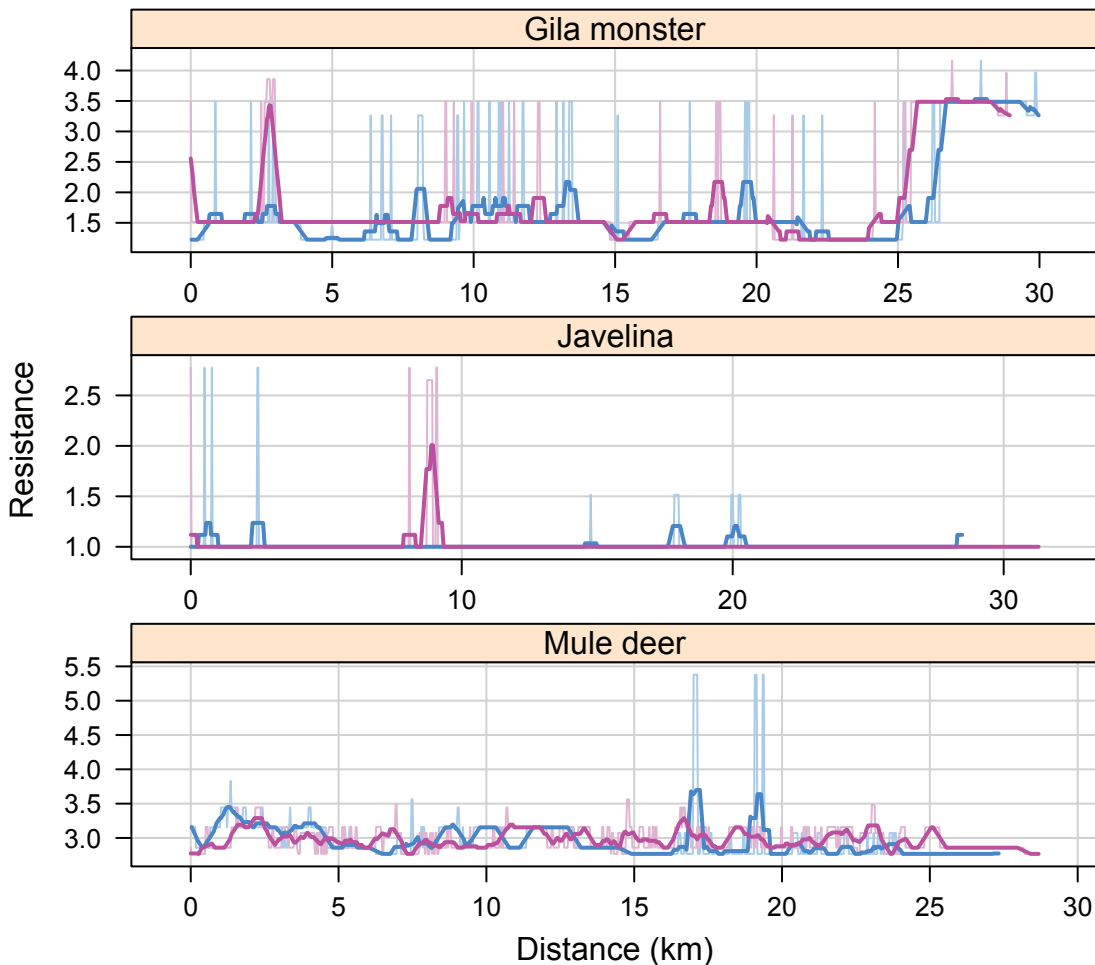

Supplement: Figure S2 — Resistance profiles for species with locally widespread habitat and desert bighorn sheep in the Wickenburg-Hassayampa planning area. The smoothed resistance profiles (in bold) are superimposed on the raw, unsmoothed profiles (thinner, fainter lines). (PDF) [file pone.0048965.s002.pdf]

Design Type  
Focal species — Land facets

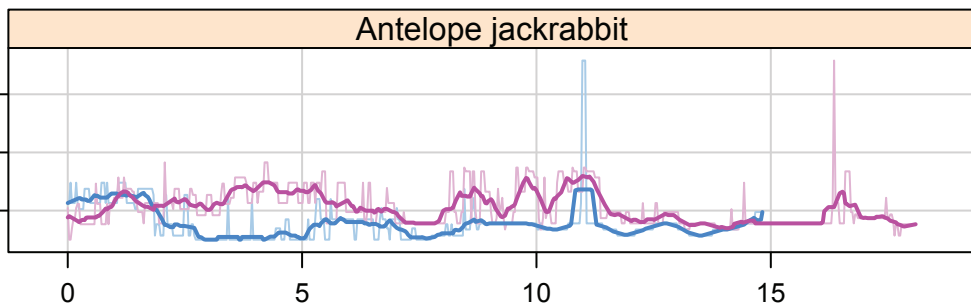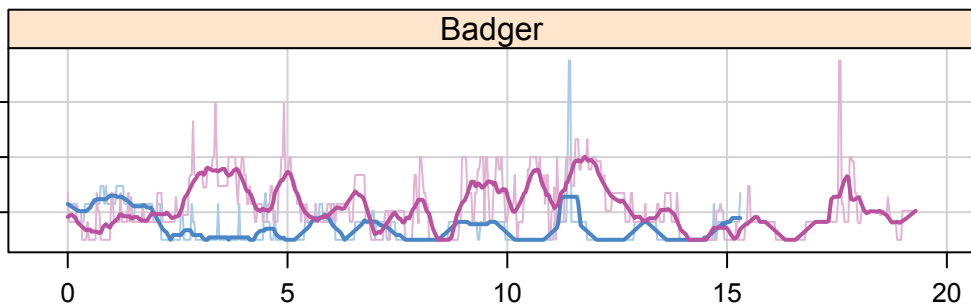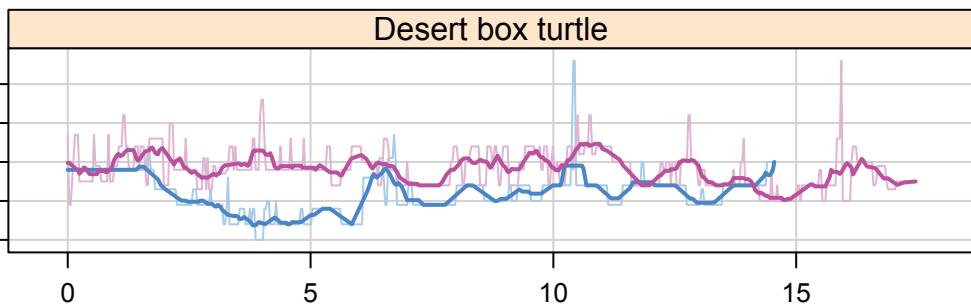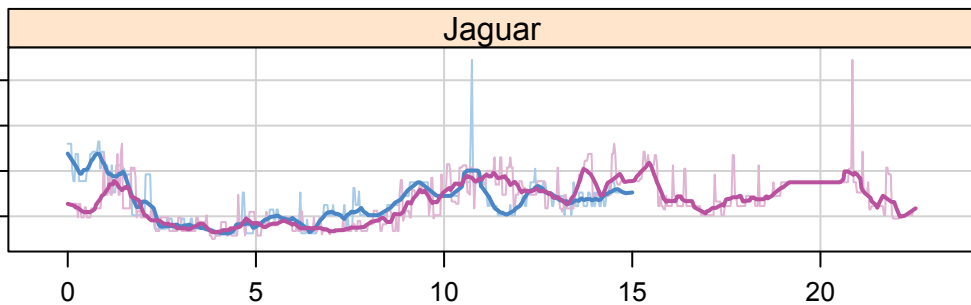

Design Type  
Focal species — Land facets

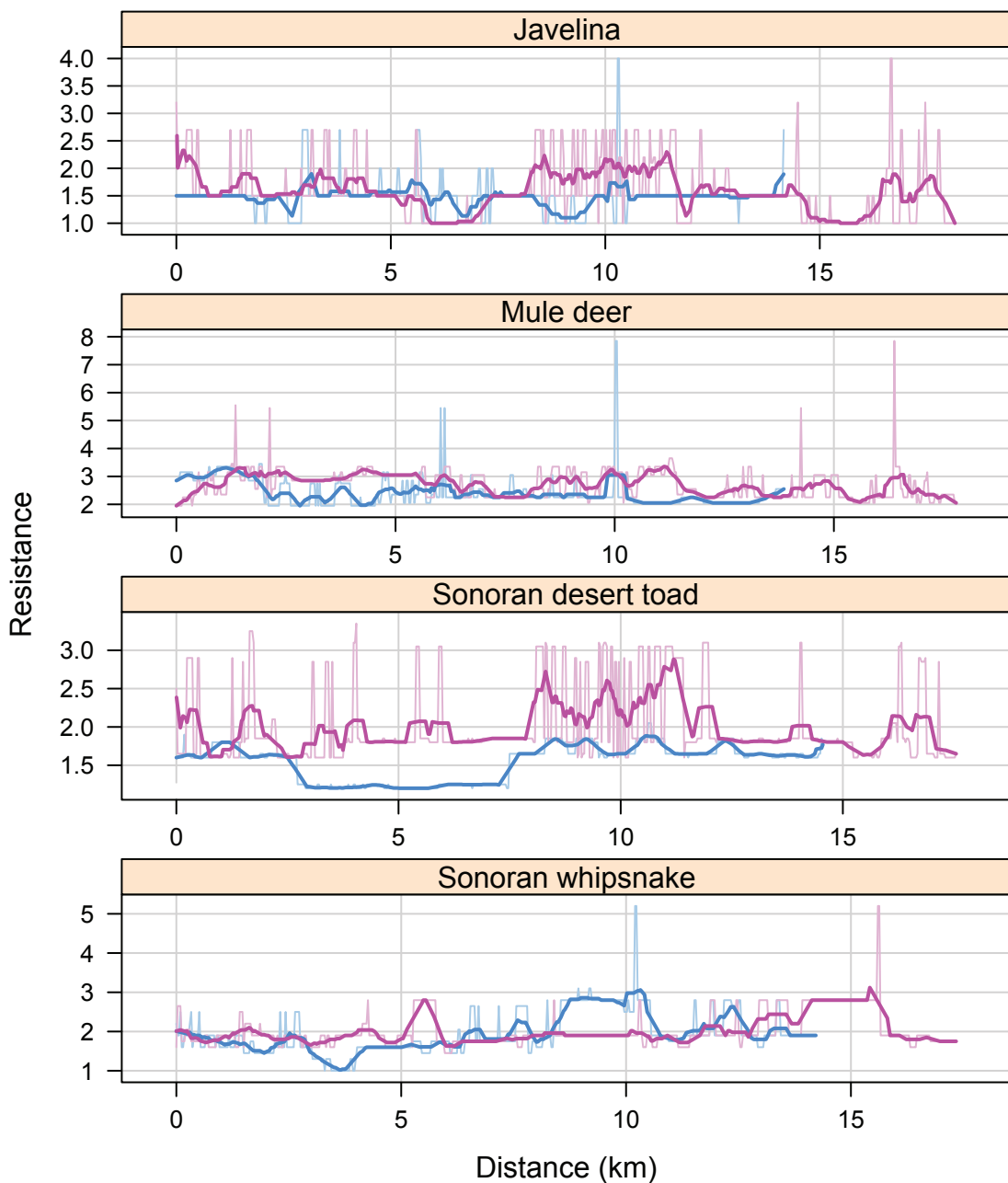

Supplement: Figure S3 — Resistance profiles for species with locally widespread habitat in the Santa Rita-Tumacacori planning area. The smoothed resistance profiles (in bold) are superimposed on the raw, unsmoothed profiles (thinner, fainter lines). (PDF) [file pone.0048965.s003.pdf]

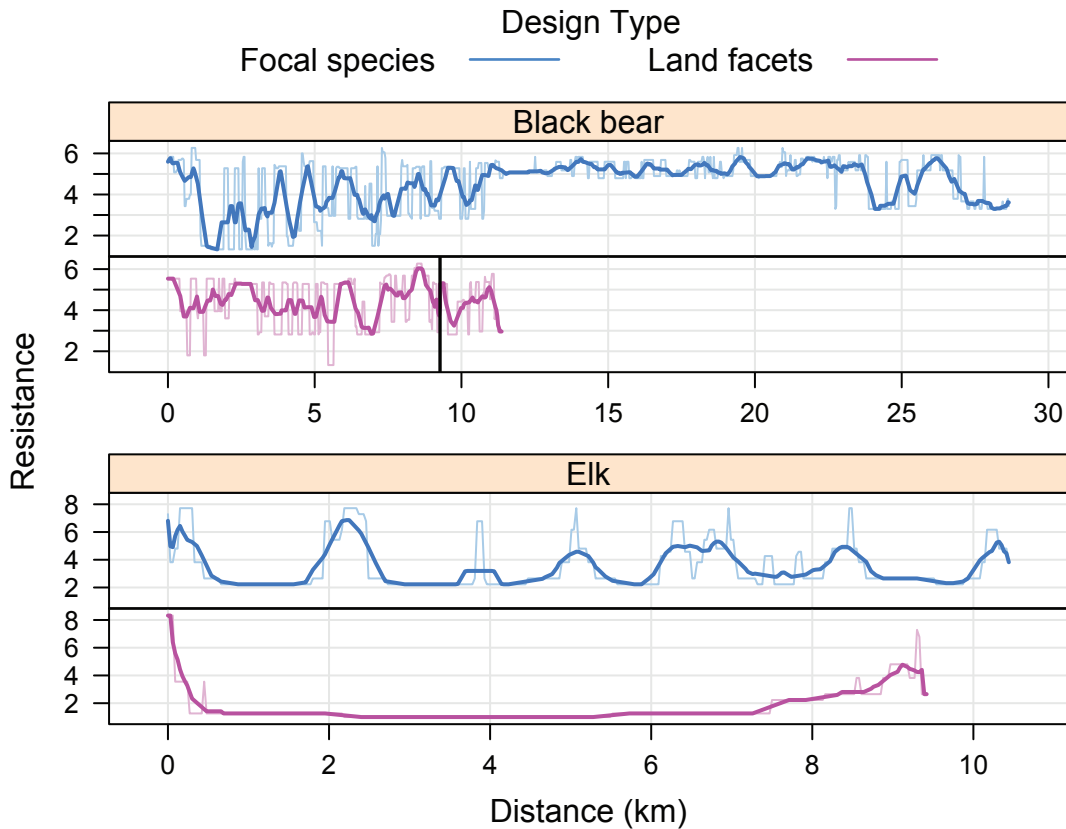

Supplement: Figure S4 — Resistance profiles corresponding to the gaps between breeding patches for black bear and elk in the Black Hills-Munds Mountain planning area. Each vertical line indicates a breeding patch between two gaps; line width does not indicate the width of the breeding patch. The smoothed resistance profiles (in bold) are superimposed on the raw, unsmoothed profiles (thinner, fainter lines). (PDF) [file pone.0048965.s004.pdf]

Design Type  
Focal species — Land facets

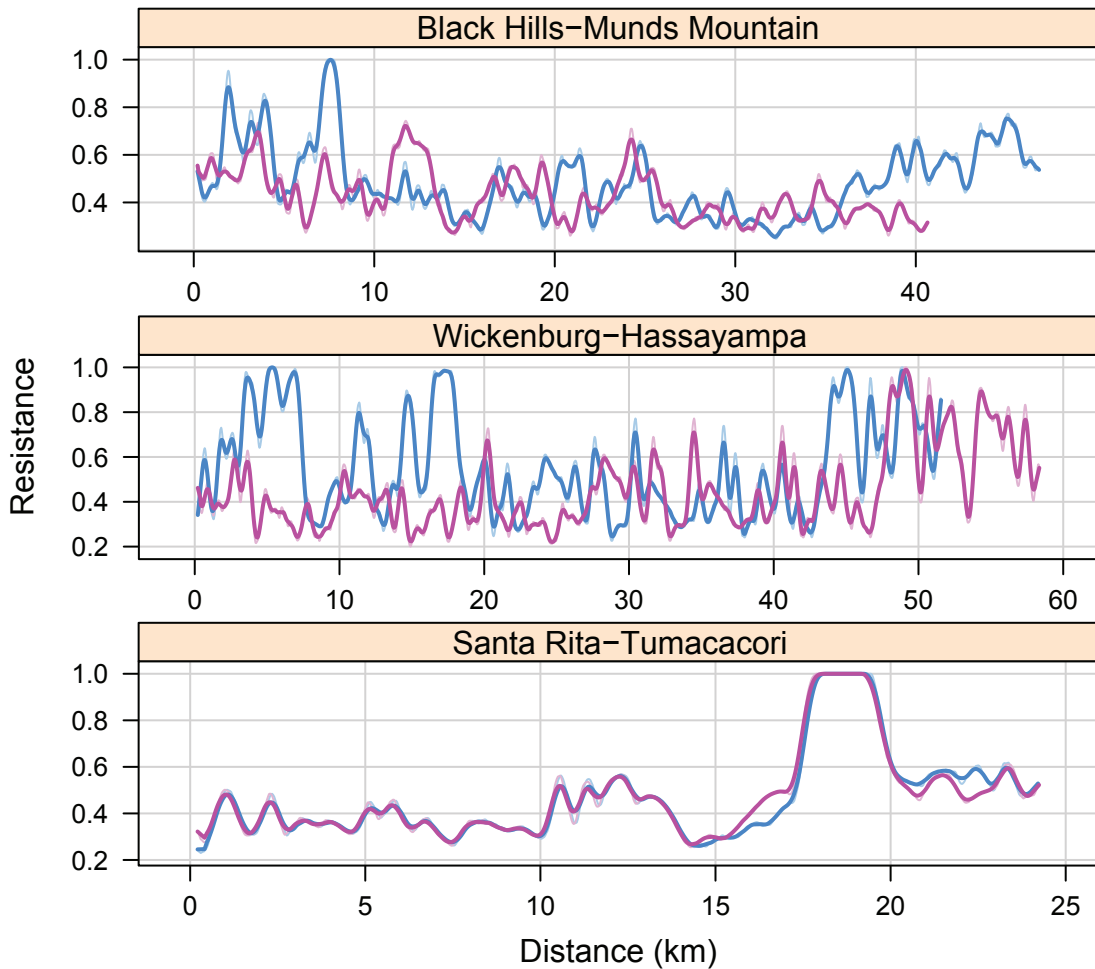

Supplement: Figure S9 — Resistance profiles for high diversity of land facets in the Black Hills-Munds Mountain, Wickenburg-Hassayampa, and Santa Rita-Tumacacori planning areas. The values in the profiles are the compliment of Shannon's evenness, where 0 is the lowest possible resistance and 1 is the maximum deviation from the optimal Shannon's index value. The smoothed resistance profiles (in bold) are superimposed on the raw, unsmoothed profiles (thinner, fainter lines). (PDF) [file pone.0048965.s009.pdf]
