# Supplementary material for: Comparing Linkage Designs Based on Land Facets to Linkage Designs Based on Focal Species
Source: PLoS One. 2012 Nov 12;7(11):e48965. doi: 10.1371/journal.pone.0048965 (PMC3495916; doi:10.1371/journal.pone.0048965)
Supplement: Figure S5 — Resistance profiles corresponding to the gaps between breeding patches for focal species in the Santa Rita-Tumacacori planning area. Each vertical line indicates a breeding patch between two gaps; line width does not indicate the width of the breeding patch. The smoothed resistance profiles (in bold) are superimposed on the raw, unsmoothed profiles (thinner, fainter lines). (PDF) [file pone.0048965.s005.pdf]

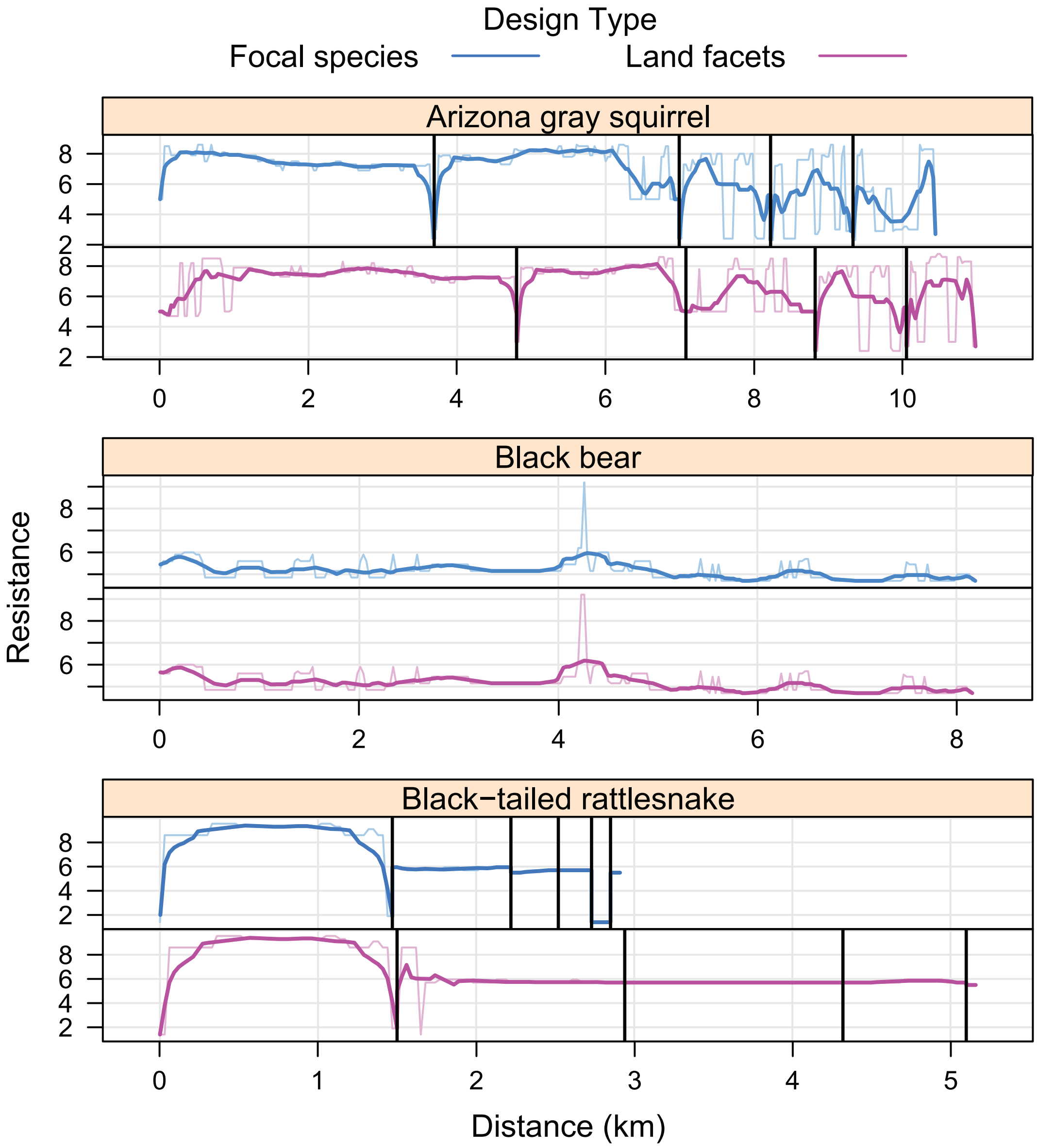
Design Type
Focal species
Land facets
Arizona gray squirrel
Black bear
Black−tailed rattlesnake
Resistance
Distance (km)
2
4
6
8
0
2
4
6
8
10
0
1
2
3
4
5

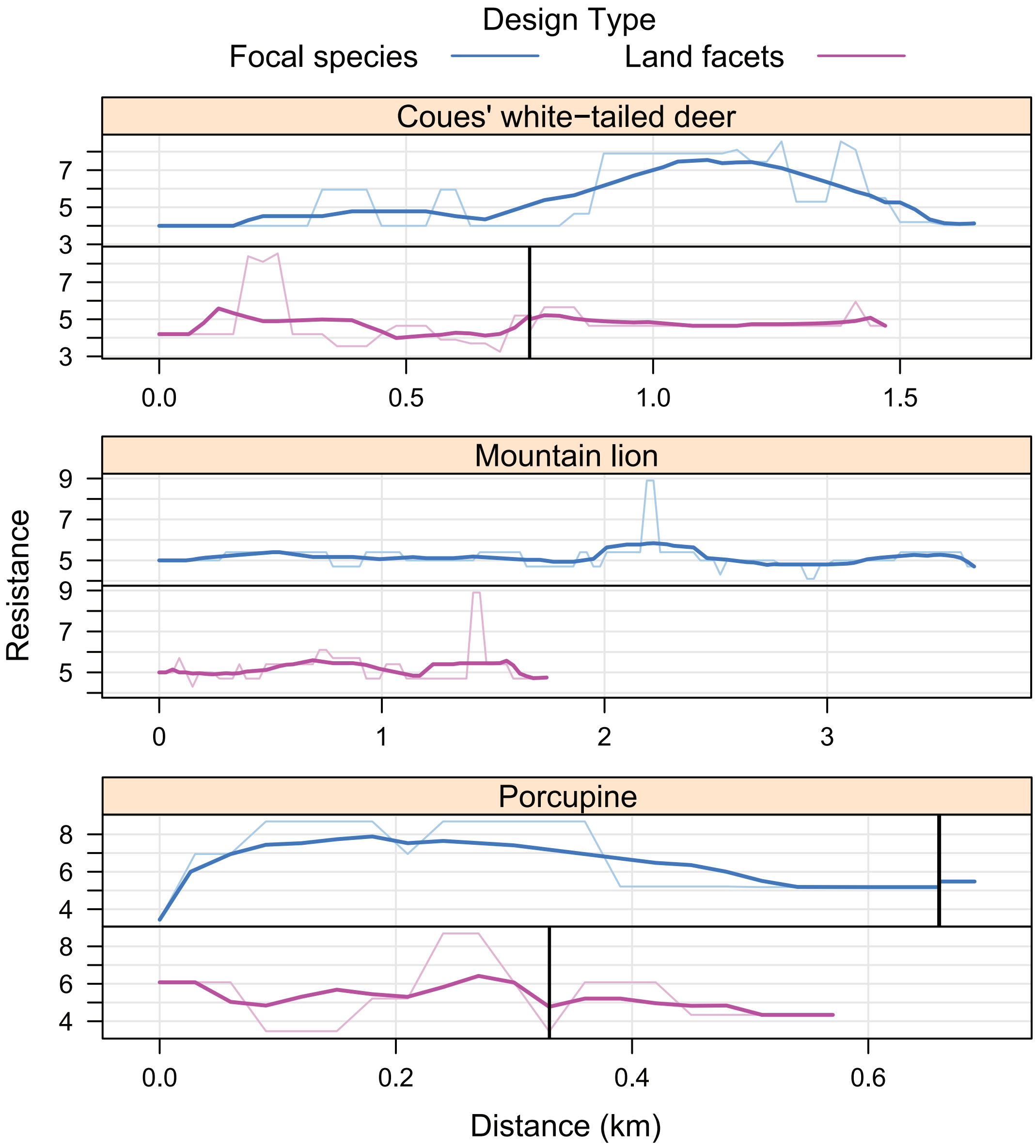
Design Type
Focal species
Land facets
Coues' white−tailed deer
Mountain lion
Porcupine
Resistance
Distance (km)

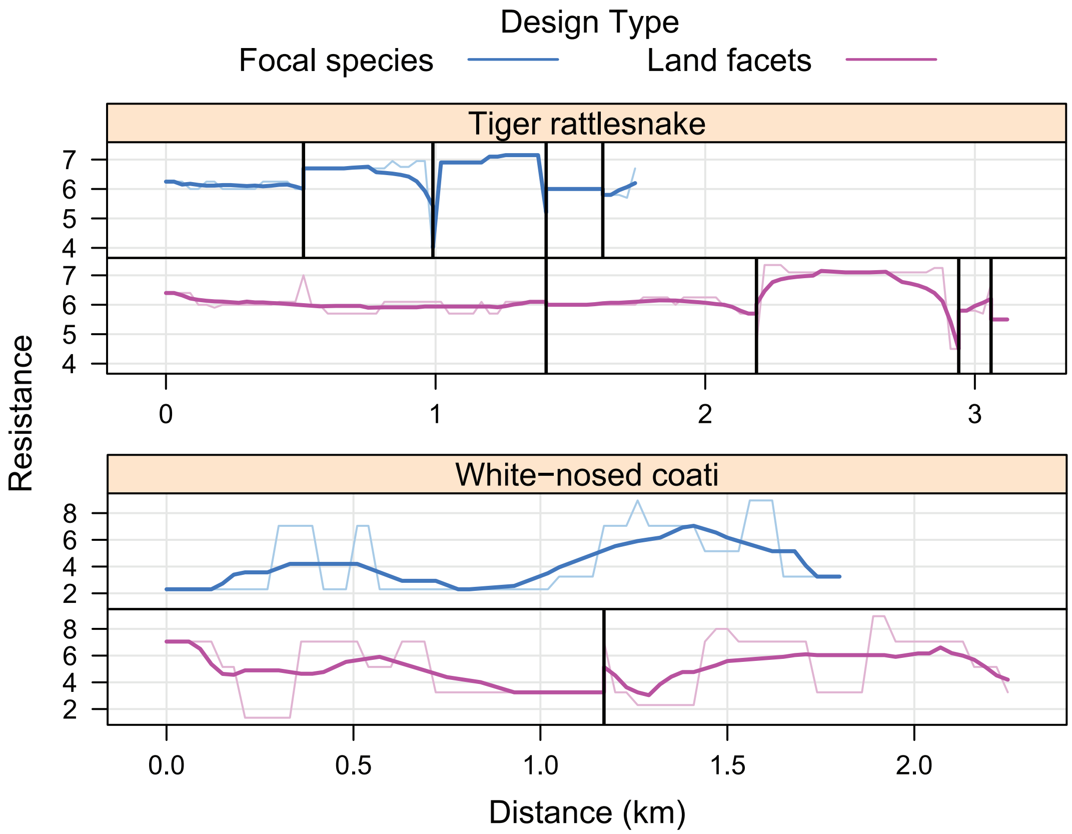

Design Type
Focal species
Land facets
Tiger rattlesnake
White−nosed coati
Resistance
Distance (km)
