# Supplementary material for: Comparing Linkage Designs Based on Land Facets to Linkage Designs Based on Focal Species
Source: PLoS One. 2012 Nov 12;7(11):e48965. doi: 10.1371/journal.pone.0048965 (PMC3495916; doi:10.1371/journal.pone.0048965)
Supplement: Figure S7 — Resistance profiles for land facets in the Wickenburg-Hassayampa planning area. The smoothed resistance profiles (in bold) are superimposed on the raw, unsmoothed profiles (thinner, fainter lines). (PDF) [file pone.0048965.s007.pdf]

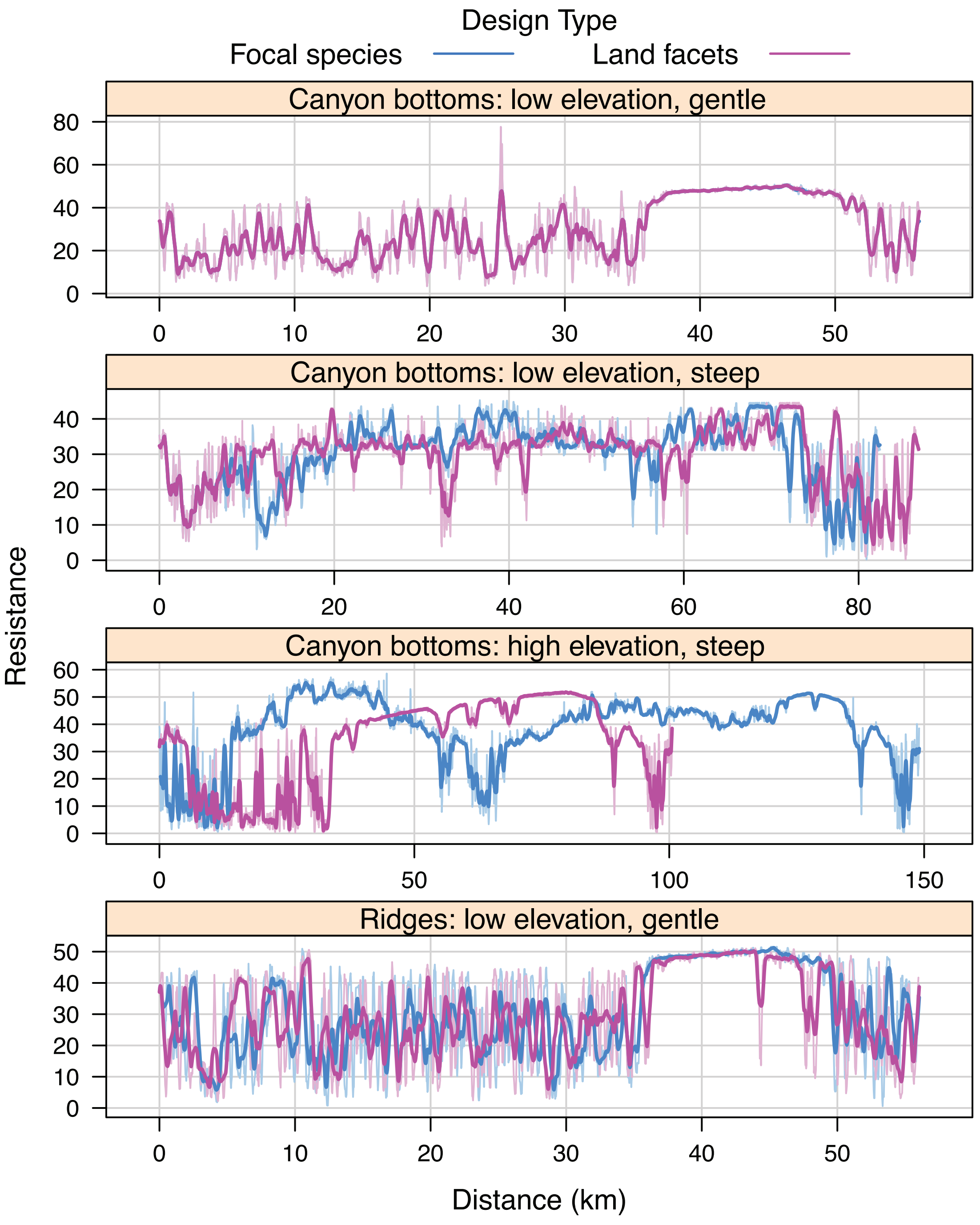

Design Type
Focal species
Land facets
Canyon bottoms: low elevation, gentle
Canyon bottoms: low elevation, steep
Canyon bottoms: high elevation, steep
Ridges: low elevation, gentle
Resistance
Distance (km)

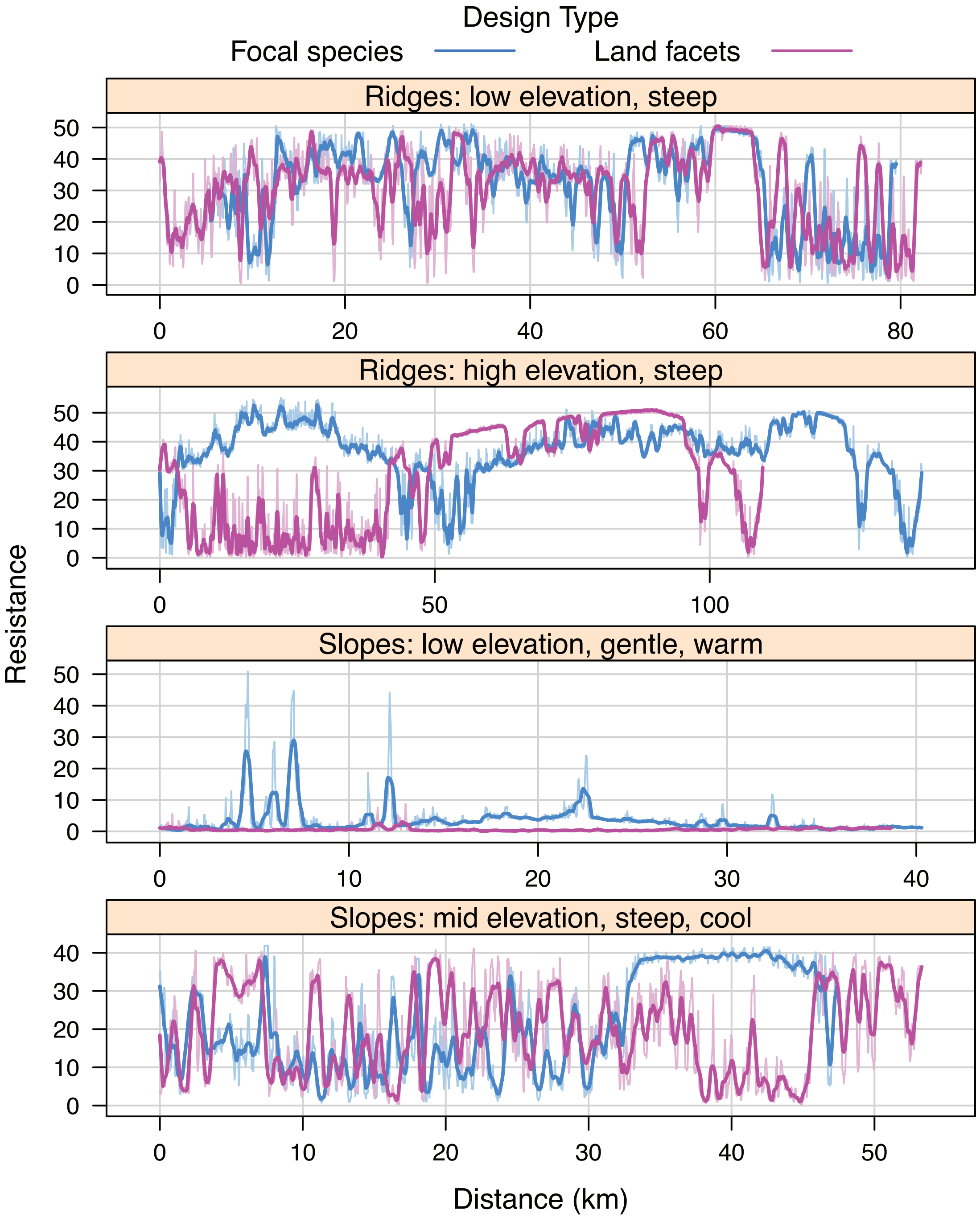
Design Type
Focal species
Land facets
Ridges: low elevation, steep
Ridges: high elevation, steep
Slopes: low elevation, gentle, warm
Slopes: mid elevation, steep, cool
Resistance
Distance (km)

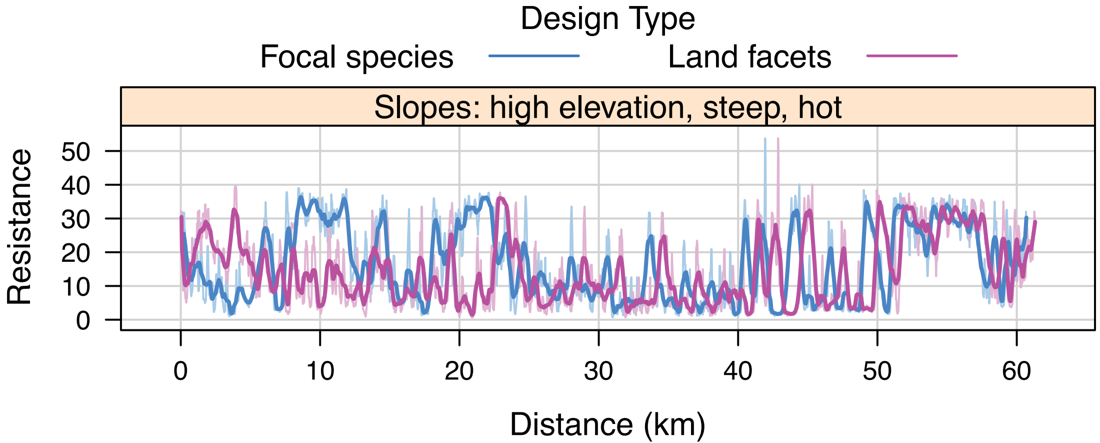
Design Type
Focal species
Land facets
Slopes: high elevation, steep, hot
Resistance
0
10
20
30
40
50
0
10
20
30
40
50
60
Distance (km)
