# Supplementary material for: Comparing Linkage Designs Based on Land Facets to Linkage Designs Based on Focal Species
Source: PLoS One. 2012 Nov 12;7(11):e48965. doi: 10.1371/journal.pone.0048965 (PMC3495916; doi:10.1371/journal.pone.0048965)
Supplement: Figure S8 — Resistance profiles for land facets in the Santa Rita-Tumacacori planning area. The smoothed resistance profiles (in bold) are superimposed on the raw, unsmoothed profiles (thinner, fainter lines). (PDF) [file pone.0048965.s008.pdf]

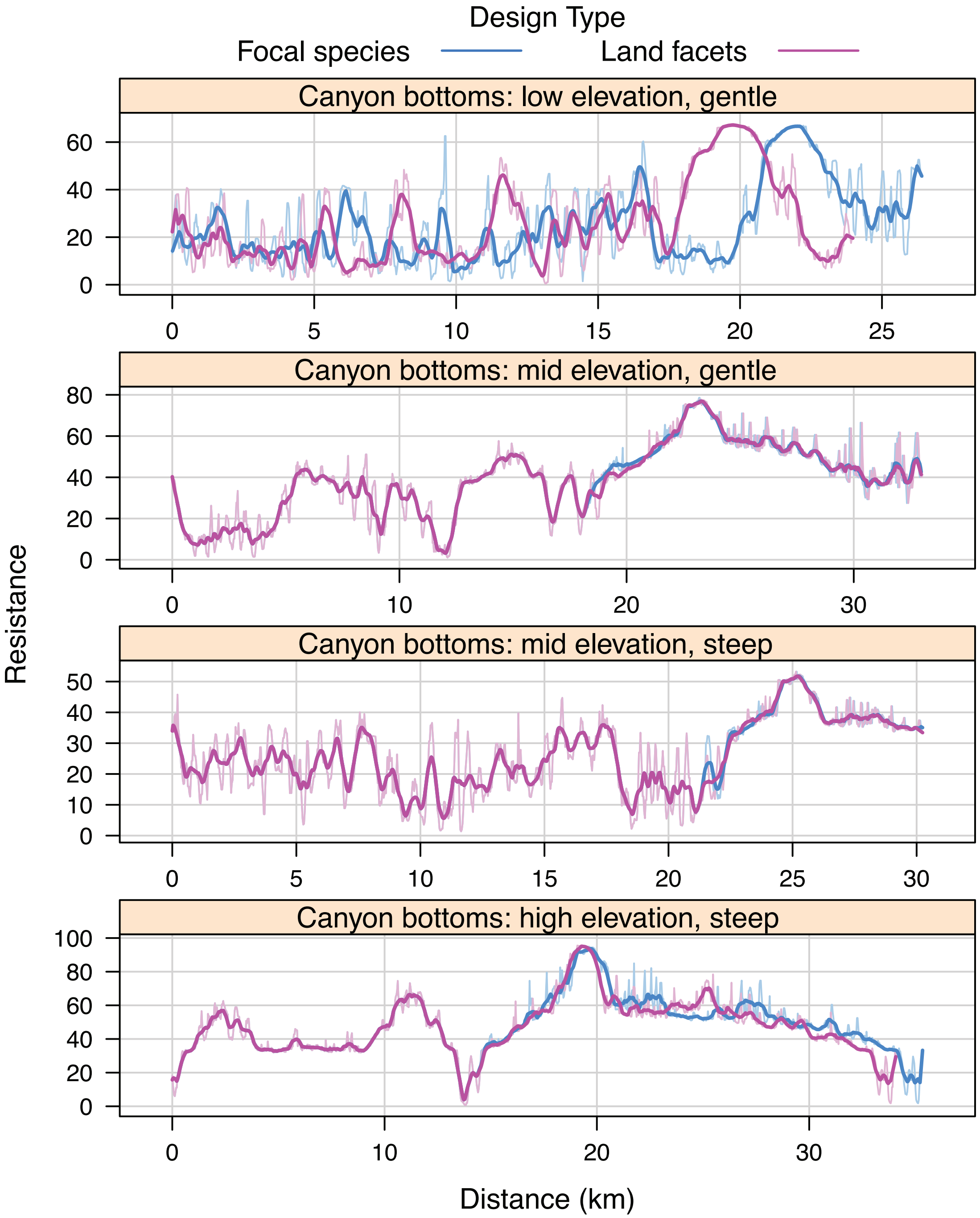

Design Type
Focal species
Land facets
Canyon bottoms: low elevation, gentle
Canyon bottoms: mid elevation, gentle
Canyon bottoms: mid elevation, steep
Canyon bottoms: high elevation, steep
Resistance
Distance (km)
0
20
40
60
80
100
0
5
10
15
20
25
30

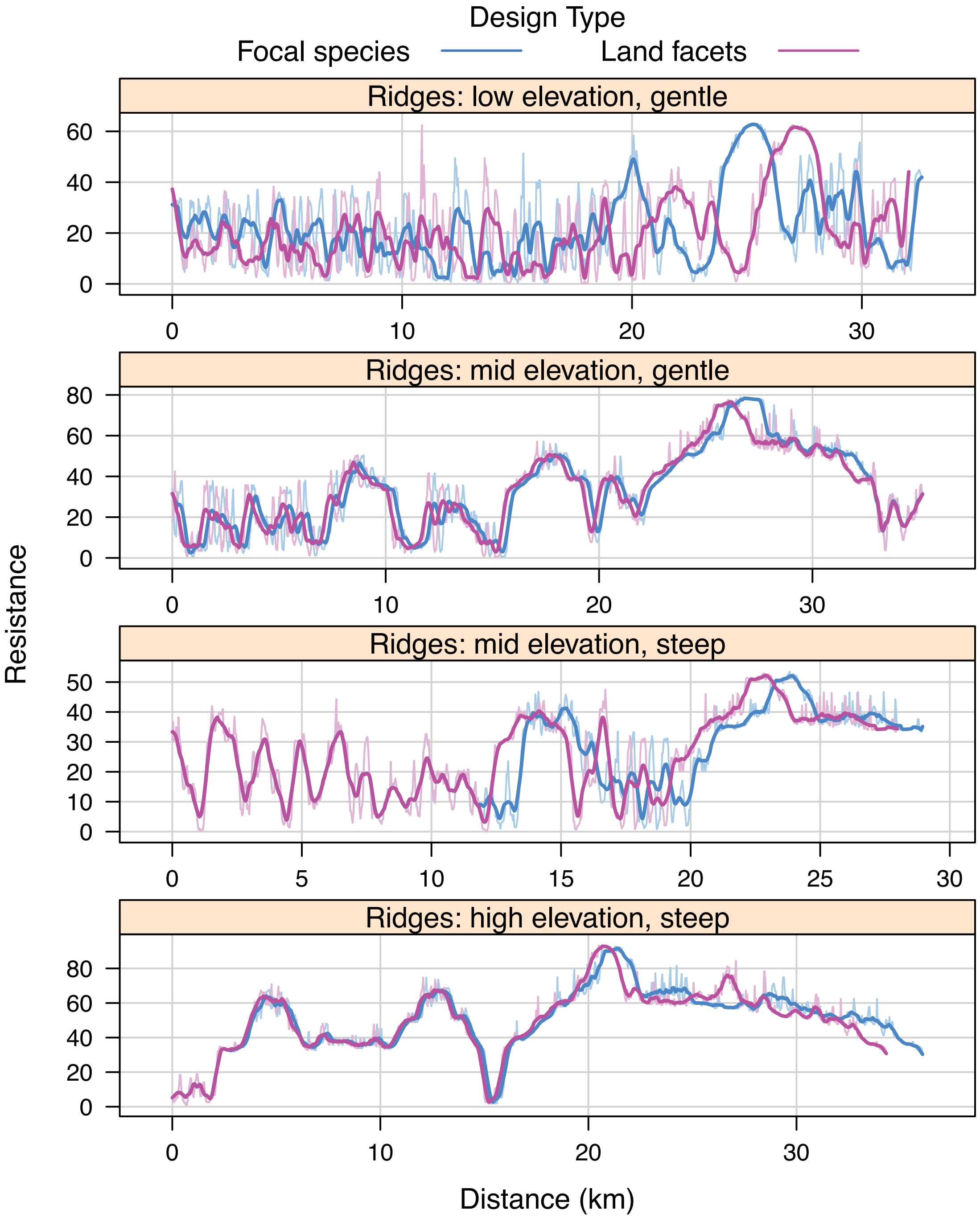

Design Type
Focal species
Land facets
Ridges: low elevation, gentle
Ridges: mid elevation, gentle
Ridges: mid elevation, steep
Ridges: high elevation, steep
Resistance
Distance (km)

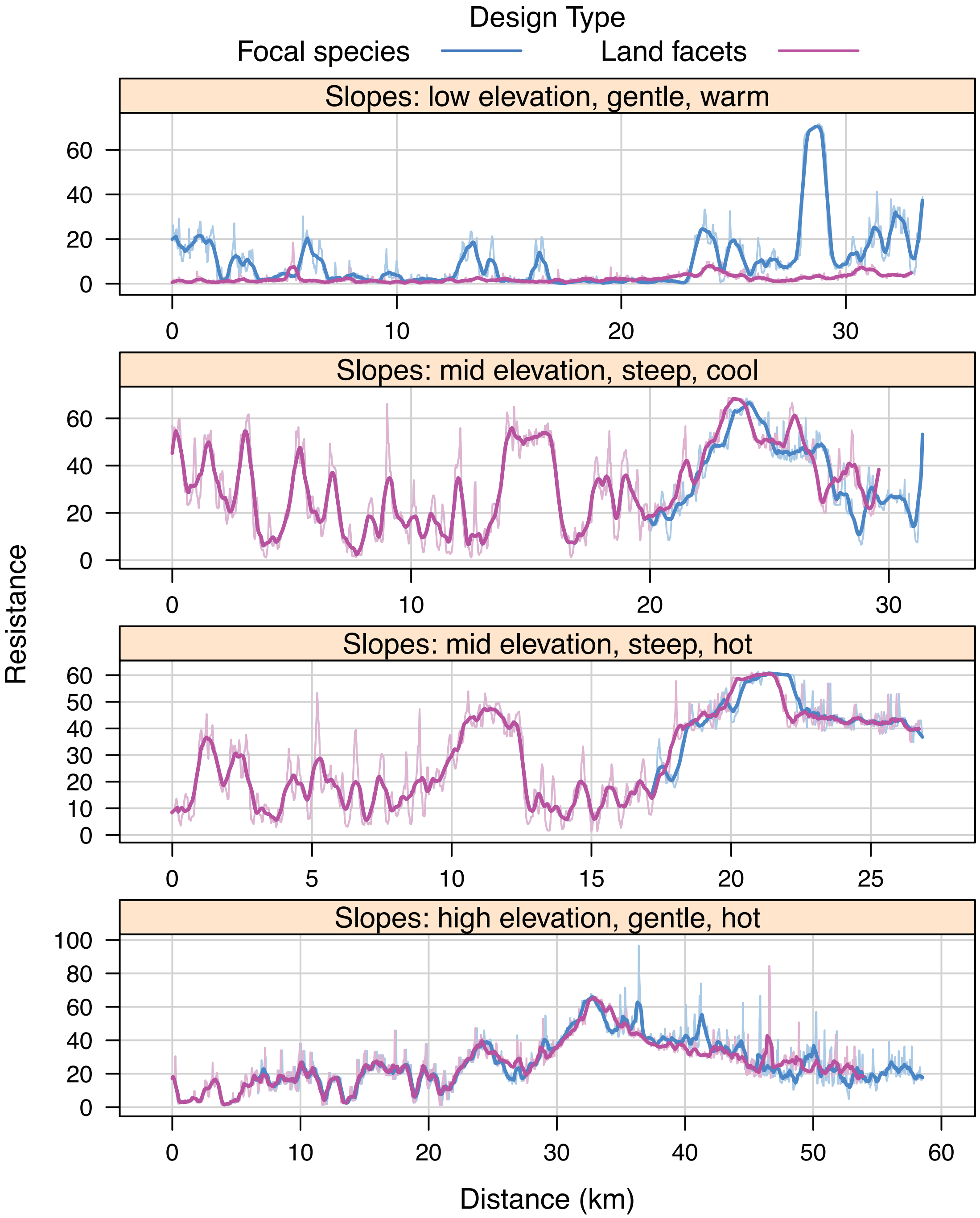

Design Type
Focal species
Land facets
Slopes: low elevation, gentle, warm
Slopes: mid elevation, steep, cool
Slopes: mid elevation, steep, hot
Slopes: high elevation, gentle, hot
Resistance
Distance (km)
